# Global burden of pancreatic cancer attributable to metabolic risks from 1990 to 2019, with projections of mortality to 2030

**DOI:** 10.1186/s12889-024-17875-6

**Published:** 2024-02-13

**Authors:** Ru He, Wenkai Jiang, Chenyu Wang, Xiao Li, Wence Zhou

**Affiliations:** The Second Clinical Medical College, Lanzhou University, No. 222 Tianshui Road (South), Cheng-Guan District, 730030 Lanzhou City, China

**Keywords:** Pancreatic cancer, Body mass index, Fasting plasma glucose, Mortality, Disability-adjusted life-year

## Abstract

**Objective:**

Metabolic risks play a key role in the progression of pancreatic cancer. This study aimed to present global, regional and national data on mortality and disability-adjusted life-year (DALY) for pancreatic cancer attributable to metabolic risk and to forecast mortality to 2030 using data from the Global Burden of Disease (GBD).

**Methods:**

Data on mortality and DALYs due to pancreatic cancer attributable to metabolic risks were obtained from GBD 2019. Metabolic risks include high fasting plasma glucose (FPG) and high body mass index (BMI). Total numbers and age-standardized rates per 100,000 people for mortality and DALYs were reported by age, sex, region and country/territory from 1990 to 2019. The “Bayes age-period-cohort” method was used for projections of mortality to 2030.

**Results:**

Globally, there was a 3.5-fold increase in the number of pancreatic cancer deaths attributable to metabolic risk, from 22,091 in 1990 to 77,215 in 2019. High-income North America and Central Europe had the highest age-standardized mortality rates (ASMRs) of pancreatic cancer attributable to high FPG and high BMI in 2019, respectively. From 1990 to 2019, the global ASMR of pancreatic cancer attributable to high FPG and high BMI increased. Countries with high healthcare access quality had much higher age-standardized DALY rates. In the next 10 years, the ASMR of pancreatic cancer attributable to high FPG and high BMI will continue to increase.

**Conclusion:**

Pancreatic cancer mortality and DALYs attributable to metabolic factors remain high, particularly in high-income regions or countries. Studies on the metabolic mechanism of pancreatic cancer and effective treatment strategies are needed.

**Supplementary Information:**

The online version contains supplementary material available at 10.1186/s12889-024-17875-6.

## Introduction

Pancreatic cancer remains one of the poorest prognoses cancers and is expected to be the second-leading cause of cancer-related death in the United States by 2030 [1]. The Global Cancer Observatory estimated that nearly 500,000 people were newly diagnosed with pancreatic cancer in 2020 worldwide, with a similar number of deaths [2]. Despite the progress made in surgery and chemotherapy during the past few decades, pancreatic cancer mortality is on the rise [3].

Modifiable risk factors, such as human behaviors and metabolic risks, play an important role in cancer progression and may be potentially preventable. There is an association between metabolism and cancer, and abnormal metabolism is a major hallmark of cancer [4, 5]. For all risk factors, metabolic risks had the greatest percentage increase in attributable cancer deaths over the past decade [6]. Obesity and diabetes are the most common metabolic disorders and contribute to many cancers. In 2019, more than 400 thousand cancer deaths were related to high body mass index (BMI) and fasting plasma glucose (FPG) worldwide [7, 8]. Population-based and experimental studies have demonstrated the relationships between metabolic factors and pancreatic cancer [3, 9]. Age-standardized pancreatic cancer deaths worldwide were primarily attributable to high FPG and high BMI in 2017, which accounted for 8.9% and 6.2%, respectively [10]. Cancer risk factors are distributed differently in different regions, and exposure to risk factors varies during different periods. In addition, the burden of metabolic risk-related disease is determined by socioeconomic, cultural, and lifestyle factors. Therefore, analyzing the epidemiological pattern of global metabolic risk-related pancreatic cancer and forecasting future trends may be beneficial for cancer prevention.

The Global Burden of Disease (GBD) study included epidemiological data on 369 diseases and 87 related risk factors, providing an opportunity to understand the epidemiological features of pancreatic cancer [11, 12]. We extracted detailed data on the deaths and disability-adjusted life years (DALYs) of pancreatic cancer attributable to metabolic risk from the GBD 2019, analyzed the temporal trend of pancreatic cancer burden, and explored the cause of its change pattern, aiming to provide new insight into this digestive system malignancy.

## Methods

### Study data

Epidemiological data were downloaded from the Global Health Data Exchange (https://ghdx.healthdata.org). We collected annual deaths, DALY counts, age-standardized mortality rates (ASMRs), age-standardized DALY rates and 95% uncertainty intervals (UIs) from the Global Burden of Disease 2019 database by year, age, sex, region and country/territory. Data were available from a total of seven GBD super-regions, 21 GBD regions, five socio-demographic index (SDI) quintiles, and all countries/territories, from 1990 to 2019.

The GBD 2019 risk factor and cause lists were organized into a hierarchy [11, 12]. In this study, we first selected “Metabolic risk” in the risk factor list. Then, “Pancreatic cancer” was selected in the list of “Neoplasms”. Identification of pancreatic cancer was based on the International Classification of Disease 10th edition using the following codes: C25-C25.9 and Z85.07 []. The general methodology for the GBD 2019 has been described previously [6, 11, 14].

Risk factor quantification was based on the comparative risk assessment framework developed by GBD Risk Factor Collaborators [12]. We identified two metabolic risk factors for pancreatic cancer: high BMI and high FPG. In the GBD 2019, high BMI was defined as BMI greater than 20 to 25 kg/m^2^, and high FPG was defined as any level above the theoretical minimum risk exposure level (4.8–5.4 mmol/L) [6]. The relative risk per five-unit change in BMI was obtained from meta-analyses, and where available, pooled analyses of prospective observational studies. For FPG, relative risks were obtained from dose-response meta-analysis of prospective cohort studies, and mortality directly caused by type 1 diabetes and type 2 diabetes was considered directly attributable to FPG [6]. The relative risk factors of high BMI and high FPG for pancreatic cancer in GBD 2019 are available at https://ghdx.healthdata.org/record/ihme-data/gbd-2019-relative-risks.

### SDI and healthcare access and quality (HAQ)

The SDI is a comprehensive index used to assess the development level of a region or country. The SDI value is computed by considering the total fertility rate among women under the age of 25, the mean educational attainment of individuals aged 15 and above, and the per capita income [15]. Countries/territories were divided into five categories by SDI values (ranging from 0 to 1: low, low-middle, middle, high-middle, and high). The HAQ, ranging from 0 to 100, is an index that estimates health-care access and quality comparable across locations [16]. The SDI and HAQ values can be extracted from GBD 2019.

### Decomposition analysis

Decomposition analysis is used to identify the factors associated with the changes in the absolute number of age-related disease burdens [17]. It can identify the additive contribution of the effect of the differences in factors between two groups (populations in 1990 and in 2019) to the difference in their overall disease burden [18]. We constructed a decomposition analysis to assess DALY changes between 1990 and 2019 by three factors: (1) age structure, (2) epidemiologic changes, and (3) population size, which can quantify the contribution of each of these factors to the overall DALY changes.

### Data analysis

Data analysis was performed in RStudio software (4.2.2). DALYs are estimated by adding years lived with disability to years of life lost [19]. One DALY represents the equivalent of one year of complete health loss [20]. All data are presented as values and their 95% UIs. All rates are reported per 100,000 people. The age-standardized rate (ASR) is a metric that can greatly eliminate the impacts of differences in age structure. The Bayesian age-period-cohort (BAPC) model can achieve more reasonable predictions and was used in previous publications [21, 22]. The “BAPC” package in RStudio software was used to predict ASR to 2030. The scatter plot is used to show the relationship between HAQ scores and ASRs.

## Results

### Burden at the global level

Overall, pancreatic cancer attributable to metabolic risks caused 77,215 (95% UI: 34,308 to 137,260) deaths worldwide in 2019, which was 3.5-fold greater than that in 1990 (22,091, 95% UI: 9,520 to 39,683). In total, pancreatic cancer attributable to metabolic risk resulted in 1,592,233 (700,905 to 2,818,765) DALYs in 2019. The global age-standardized mortality rate (ASMR) was 0.61 (95% UI: 0.14 to 1.3) for high FPG and 0.40 (95% UI: 0.15 to 0.74) for high BMI in 2019 (Tables 1 and 2). Over the past 30 years, the ASMRs attributable to high FPG and high BMI increased by 66.9% (56.2 to 83) and 51.6% (36.2 to 72), respectively. Like those of the ASMR, the age-standardized DALY rates also increased from 1990 to 2019.

**Table 1:** Age-standardized mortality and DALY rates of pancreatic cancer attributable to high fasting plasma glucose in 2019 and their percentage changes from 1990 to 2019 in different regions

Location	ASMR in 2019 (per 100,000)	Percentage change of ASMR, 1990 to 2019 (%)	Age-standardized DALY rate in 2019 (per 100,000)	Percentage change of age-standardized DALY rate, 1990 to 2019 (%)
Global	0.61 (0.14 to 1.3)	66.9 (56.2 to 83)	11.49 (2.7 to 24.74)	66.1 (54.8 to 82.1)
High SDI	0.97 (0.23 to 2.07)	59.6 (49.8 to 75.3)	18.56 (4.36 to 39.59)	55.8 (47 to 70)
High-middle SDI	0.65 (0.15 to 1.41)	65.6 (52.8 to 83.1)	12.68 (2.89 to 27.91)	62 (49.3 to 79.7)
Middle SDI	0.44 (0.1 to 0.95)	117.4 (93.5 to 147.5)	8.7 (1.97 to 18.96)	116.1 (91.3 to 148)
Low-middle SDI	0.35 (0.08 to 0.75)	194.3 (151.6 to 266.3)	7.02 (1.63 to 15.31)	194.7 (149.5 to 268.1)
Low SDI	0.22 (0.05 to 0.49)	131.6 (93.4 to 186.2)	4.39 (1 to 9.95)	131.2 (91 to 188.3)
Andean Latin America	0.47 (0.11 to 1.06)	314.9 (241.4 to 412.9)	8.57 (1.93 to 19.63)	300.2 (225.8 to 402.2)
Australasia	0.63 (0.14 to 1.4)	71.9 (53.7 to 96.2)	11.16 (2.49 to 24.82)	71.5 (55.1 to 95.2)
Caribbean	0.65 (0.16 to 1.35)	311.4 (252.7 to 386.5)	12.63 (2.99 to 26.84)	310.7 (249.1 to 388.3)
Central Asia	0.54 (0.12 to 1.16)	357 (288.9 to 463.2)	11.07 (2.56 to 24.22)	337.7 (276 to 433.9)
Central Europe	1.06 (0.25 to 2.31)	77.4 (54.5 to 106.3)	21.88 (5 to 48.06)	76.5 (53 to 105.8)
Central Latin America	0.73 (0.18 to 1.56)	29.2 (11.9 to 48.5)	14.76 (3.53 to 31.84)	34.8 (16.6 to 55.7)
Central Sub-Saharan Africa	0.25 (0.06 to 0.57)	63.3 (24.5 to 119.2)	5.28 (1.15 to 12.05)	65.8 (23.7 to 123.5)
East Asia	0.42 (0.09 to 0.95)	109.4 (73.2 to 158.1)	8.58 (1.87 to 19.67)	101.3 (63.8 to 150.8)
Eastern Europe	0.42 (0.1 to 0.94)	63.3 (45.8 to 85.4)	9.06 (2.06 to 20.56)	58.8 (41.2 to 81)
Eastern Sub-Saharan Africa	0.19 (0.04 to 0.43)	96.9 (60.7 to 142.9)	3.76 (0.82 to 8.6)	100.4 (61.8 to 152.8)
High-income Asia Pacific	0.65 (0.15 to 1.47)	18.7 (9.5 to 26.3)	11.97 (2.66 to 26.8)	12.1 (5.5 to 18.9)
High-income North America	1.22 (0.3 to 2.55)	67 (56 to 86.7)	23.82 (5.67 to 49.76)	59.8 (49.5 to 77.3)
North Africa and Middle East	0.62 (0.15 to 1.33)	224 (155.7 to 336)	12.43 (2.95 to 27.02)	218.7 (154.3 to 322.6)
Oceania	0.38 (0.1 to 0.82)	108.1 (69.6 to 167.4)	8.22 (1.98 to 18.17)	112.6 (72.1 to 176.2)
South Asia	0.31 (0.07 to 0.67)	206.8 (147.4 to 299.8)	6.2 (1.46 to 13.63)	206.7 (143.6 to 302.5)
Southeast Asia	0.41 (0.09 to 0.9)	181.6 (129.3 to 252.9)	7.66 (1.76 to 17.34)	172 (121.4 to 240.1)
Southern Latin America	1.04 (0.25 to 2.25)	91.8 (69.3 to 123)	19.88 (4.66 to 43.06)	89.4 (66.6 to 119.5)
Southern Sub-Saharan Africa	0.68 (0.16 to 1.45)	100.9 (69 to 139.8)	13.02 (3.08 to 28.2)	109.9 (78.8 to 151)
Tropical Latin America	0.59 (0.14 to 1.28)	34.8 (25 to 45.4)	11.41 (2.69 to 24.71)	32.1 (23 to 42.6)
Western Europe	0.99 (0.24 to 2.1)	66.8 (55.5 to 84.9)	18.26 (4.36 to 39.14)	63.5 (52.7 to 79.9)
Western Sub-Saharan Africa	0.36 (0.08 to 0.8)	199.4 (134.7 to 286.7)	6.71 (1.51 to 15.13)	200.2 (135 to 291.9)

ASMR: age-standardized mortality rate; DALY: disability-adjusted life year; SDI: socio-demographic index

**Table 2:** Age-standardized mortality and DALY rates of pancreatic cancer attributable to high body-mass index in 2019 and their percentage changes from 1990 to 2019 in different regions

Location	ASMR in 2019 (per 100,000)	Percentage change of ASMR, 1990 to 2019 (%)	Age-standardized DALY rate in 2019 (per 100,000)	Percentage change of age-standardized DALY rate, 1990 to 2019 (%)
Global	0.4 (0.15 to 0.74)	51.6 (36.2 to 72)	8.54 (3.09 to 15.99)	52.6 (37.1 to 73.4)
High SDI	0.67 (0.25 to 1.24)	42.5 (25.9 to 64.9)	14.62 (5.23 to 27.31)	41 (26.2 to 63.4)
High-middle SDI	0.52 (0.19 to 0.96)	48.9 (32.6 to 69.2)	11.64 (4.15 to 21.94)	45.3 (29.1 to 65.5)
Middle SDI	0.24 (0.09 to 0.47)	186 (136.8 to 270.5)	5.74 (2.11 to 11.23)	181.6 (132.9 to 265.6)
Low-middle SDI	0.15 (0.06 to 0.3)	307 (204.3 to 513.8)	3.57 (1.32 to 6.92)	309.7 (206.5 to 511.2)
Low SDI	0.09 (0.03 to 0.18)	199.2 (124.9 to 361.9)	2.18 (0.73 to 4.42)	196 (120.3 to 355.2)
Andean Latin America	0.43 (0.17 to 0.79)	315.1 (220.3 to 486.7)	9.88 (3.89 to 17.84)	285.2 (194.4 to 432)
Australasia	0.69 (0.26 to 1.23)	46 (28 to 75.9)	14.67 (5.46 to 26.32)	41.2 (24.5 to 69.1)
Caribbean	0.39 (0.15 to 0.72)	345.9 (263.4 to 455.5)	8.9 (3.32 to 16.61)	334.2 (246.8 to 441)
Central Asia	0.48 (0.2 to 0.86)	221.5 (161.4 to 305.1)	11.11 (4.23 to 20.29)	204.7 (147.5 to 280.1)
Central Europe	0.97 (0.36 to 1.74)	54.7 (31.8 to 80.2)	22.2 (7.96 to 40.98)	49.3 (26.4 to 74.6)
Central Latin America	0.43 (0.17 to 0.78)	57.2 (31.6 to 91.8)	9.88 (3.84 to 18.17)	53.4 (27.8 to 86.8)
Central Sub-Saharan Africa	0.1 (0.03 to 0.21)	72.6 (23.3 to 162.5)	2.44 (0.78 to 5.28)	71.4 (20.3 to 163.8)
East Asia	0.21 (0.06 to 0.48)	276.1 (167.7 to 599.6)	5.11 (1.31 to 11.49)	263 (153.6 to 587.9)
Eastern Europe	0.75 (0.3 to 1.34)	54.6 (28.5 to 85.9)	18 (6.9 to 32.81)	50 (24 to 81.2)
Eastern Sub-Saharan Africa	0.11 (0.04 to 0.23)	217.9 (131.9 to 427)	2.8 (1.01 to 5.67)	214.2 (126.1 to 429.7)
High-income Asia Pacific	0.28 (0.07 to 0.64)	20.6 (6.3 to 48.8)	5.7 (1.46 to 13.15)	13.4 (1.2 to 37.9)
High-income North America	0.91 (0.35 to 1.62)	51.5 (33.9 to 78.4)	20.03 (7.5 to 35.5)	45.8 (29.9 to 70.4)
North Africa and Middle East	0.48 (0.18 to 0.88)	189.3 (115.7 to 293.9)	11.26 (4.07 to 20.75)	177.5 (110.7 to 277.5)
Oceania	0.14 (0.05 to 0.28)	67.3 (35.7 to 115.1)	3.35 (1.11 to 6.82)	62.1 (30.4 to 108.7)
South Asia	0.1 (0.04 to 0.2)	424.3 (254.6 to 889)	2.41 (0.86 to 4.6)	421.6 (252.8 to 855.6)
Southeast Asia	0.16 (0.06 to 0.33)	329.9 (189.9 to 665)	4.02 (1.43 to 7.97)	304.6 (175.8 to 601.4)
Southern Latin America	0.8 (0.31 to 1.46)	77.4 (49.1 to 131.7)	17.12 (6.47 to 31.99)	71.6 (44.5 to 121.2)
Southern Sub-Saharan Africa	0.57 (0.24 to 0.98)	86.8 (55.9 to 125.8)	12.58 (5.2 to 21.9)	77.3 (51.2 to 110.4)
Tropical Latin America	0.5 (0.2 to 0.9)	87 (57.1 to 139.8)	11.18 (4.29 to 20.31)	79.9 (52.4 to 127.8)
Western Europe	0.67 (0.24 to 1.25)	40.4 (27.5 to 57.6)	14.1 (4.97 to 26.66)	35.3 (24.2 to 51.8)
Western Sub-Saharan Africa	0.26 (0.1 to 0.5)	310.5 (189.5 to 533.1)	6.12 (2.46 to 11.6)	295.1 (182.4 to 508.8)

ASMR: age-standardized mortality rate; DALY: disability-adjusted life year; SDI: socio-demographic index

**Fig. 1:**
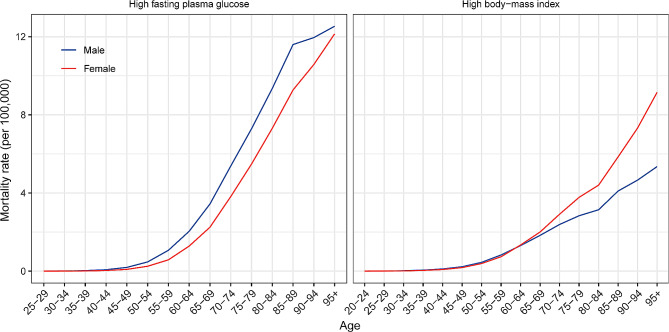
The global age-specific mortality rates of pancreatic cancer attributable to high fasting plasma glucose and high body-mass index by sex in 2019

In 2019, the mortality rate increased with age (Fig. 1). People older than 95 years had the highest mortality rate for both sexes. The mortality rates of pancreatic cancer attributed to high FPG were higher in males than in females in all age groups. Among people under 60 years of age, males had higher mortality rates of pancreatic cancer attributed to high BMI than females in the same age group, while females had higher mortality rates than males among people over 60 years old. The age-specific DALY rates in the different age groups are shown in Figure S1.

### Burden at the regional and national levels

**Fig. 2:**
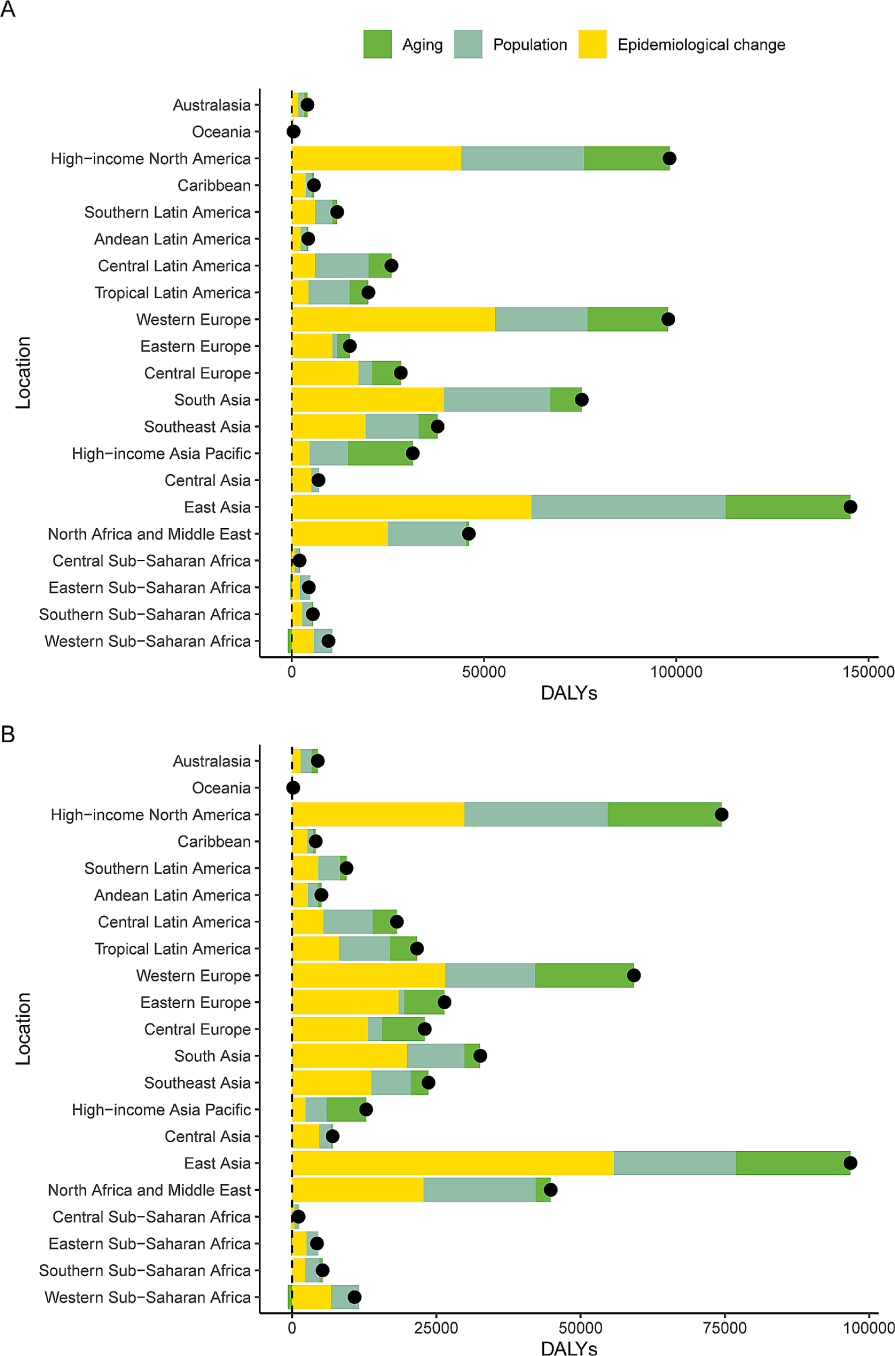
Decomposition analysis of changes in the DALY number of pancreatic cancer attributable to (**A**) high fasting plasma glucose and (**B**) high body-mass index between 1990 and 2019 across 21 GBD regions. DALY: disability-adjusted life year

The three GBD regions with the highest ASMRs of pancreatic cancer attributed to metabolic risk were high-income North America, Central Europe and Southern Latin America in 2019 (Figure S2). Among the 21 GBD regions, for the increase in DALYs caused by high FPG, the contribution of age structure was the highest in high-income Asia Pacific (53.53%), and the contribution of population growth was highest in Central Sub-Saharan Africa (68.12%) (Fig. 2A). For DALYs caused by high BMI, high-income Asia Pacific and Central Sub-Saharan Africa also had the highest contributions to age structure and population growth, respectively (Fig. 2B). The proportions of pancreatic cancer mortality attributable to high BMI and high FPG among 21 GBD regions in 2019 are shown in Figure S3.

**Fig. 3:**
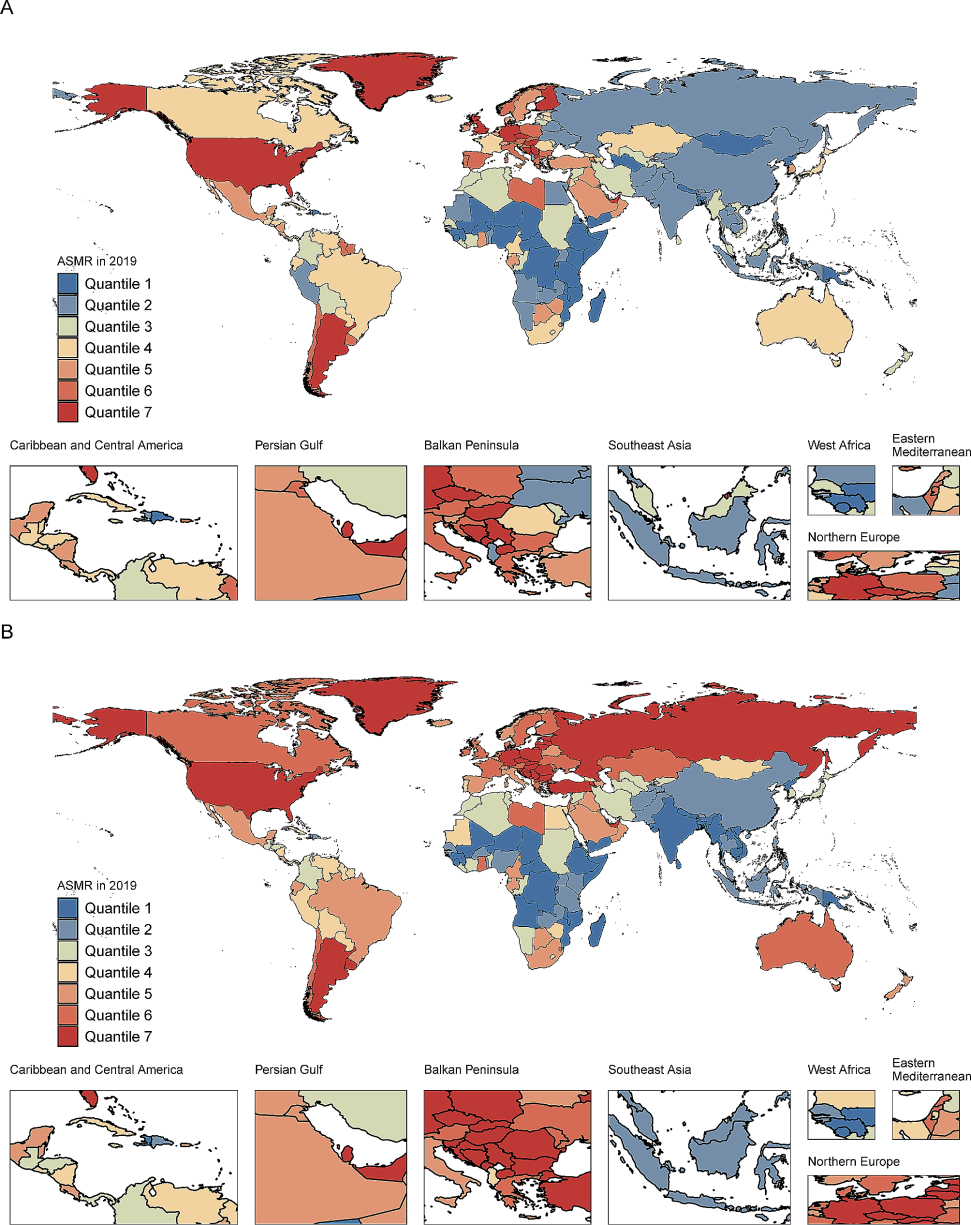
Age-standardized mortality rate of pancreatic cancer attributable to (**A**) high fasting plasma glucose and (**B**) high body-mass index in all countries/territories in 2019. ASMR: age-standardized mortality rate

At the national level, the United Arab Emirates had the highest ASMR for high FPG in 2019, followed by Qatar and Palau. The three countries with the highest ASMR for high BMI in 2019 were United Arab Emirates, Greenland and Monaco (Fig. 3). Over the past 30 years, Cabo Verde and Equatorial Guinea had the highest increases in the ASMR for high FPG and high BMI, respectively. No country/territory showed a decreasing trend in the ASMR for pancreatic cancer attributable to metabolic risks. The counts and ASRs for pancreatic cancer mortality and DALYs attributable to metabolic risk in 204 countries/territories in 1990 and 2019 are shown in Supplementary Tables S1 and S2.

### Burden at different SDI levels

**Fig. 4:**
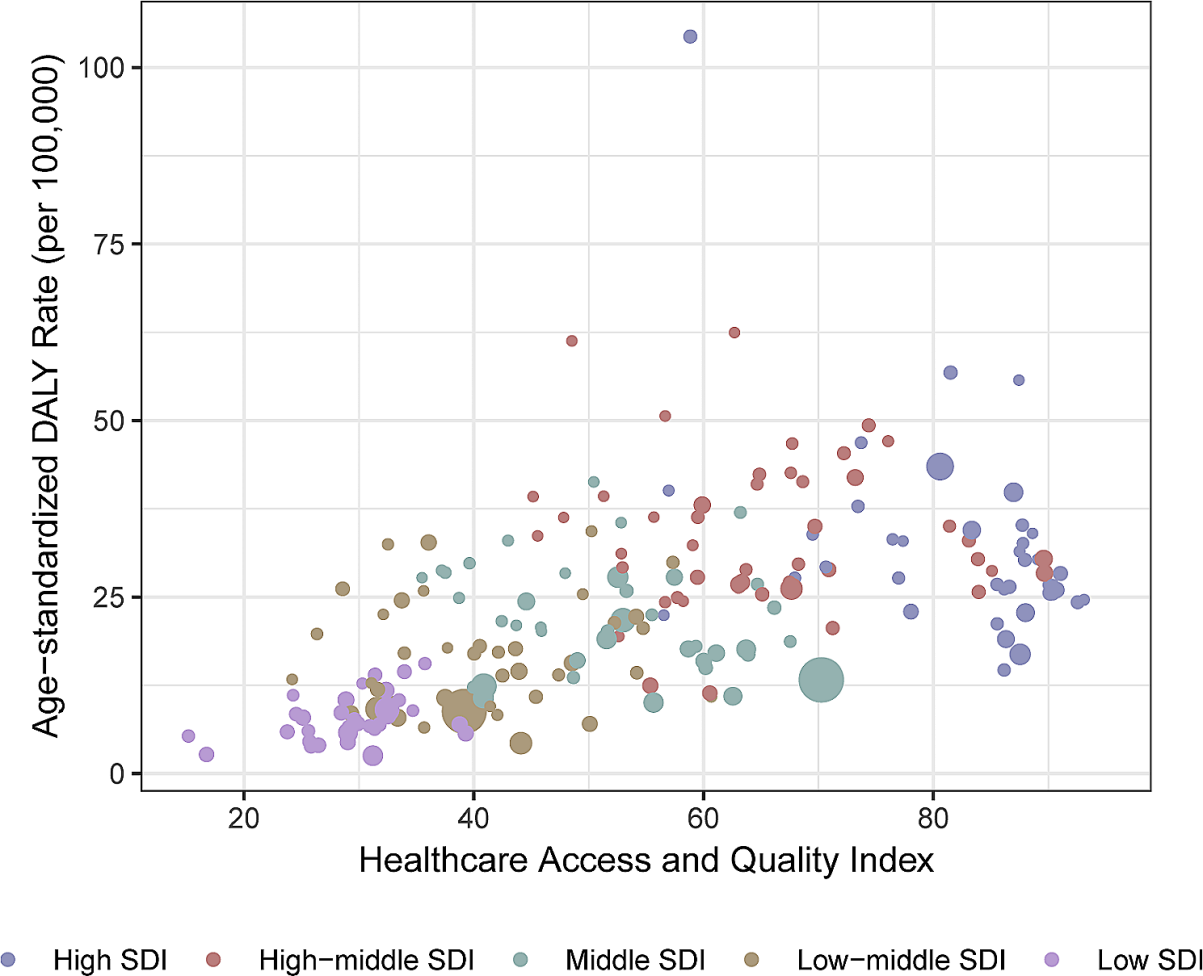
Association between age-standardized DALY rate and healthcare access and quality index in 2019. DALY: disability-adjusted life year

The age-standardized mortality and DALY rates of pancreatic cancer attributable to metabolic risks were consistently highest in high-SDI regions in 1990 and 2019 (Figures S4 and S5). The percentage change in the ASMR was highest in the low-middle SDI quintile (217%) and lowest in the high SDI quintile (50%). In 2019, the ASMR of pancreatic cancer attributable to metabolic risks increased as SDI increased, with the highest occurring in the high-SDI quintile (1.57, 95% UI: 0.7 to 2.76) and gradually decreasing to the lowest level occurring in the low-SDI quintile (0.3, 95% UI: 0.11 to 0.59). Figure 4 shows the distribution of age-standardized DALY rates in relation to countries’ HAQ. Countries with high HAQ scores had higher age-standardized DALY rates, whereas countries with low HAQ experienced significantly reduced age-standardized DALY rates. The relationship between the ASMR and HAQ is shown in Figure S6.

Figure S7 and S8 present the relationships between age-standardized mortality and DALY rates and SDIs in 21 GBD regions during the past 30 years. The ASRs in all GBD regions increased as the SDI increased from 1990 to 2019. In high-income regions, the ASRs of high-income North America continue to increase and are still well above the fitted curve in many years, while high-income Asia Pacific and Australasia are lower than the fitted curve.

### Projection of mortality to 2030

**Fig. 5:**
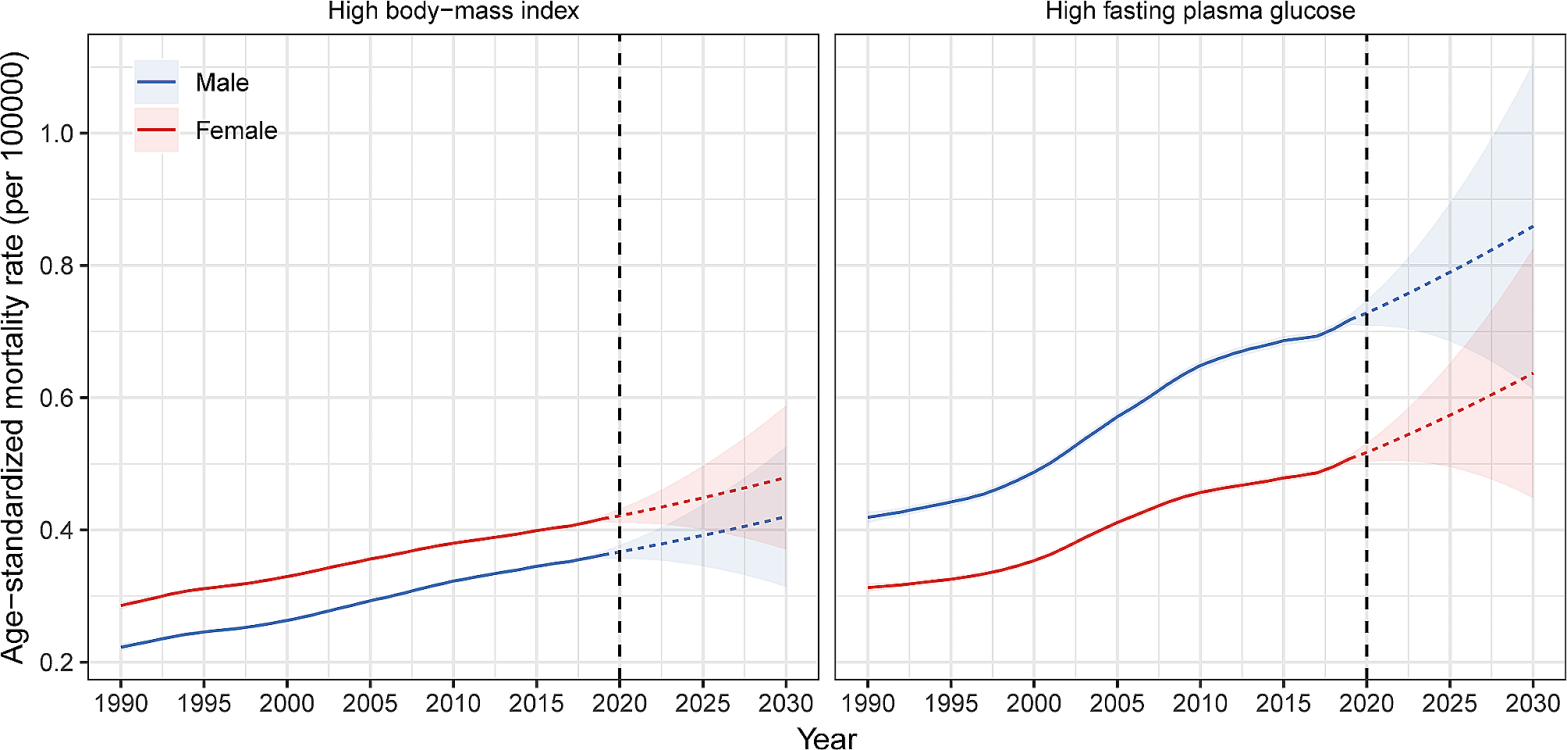
Time trend of global age-standardized mortality rate of pancreatic cancer attributable to (**A**) high fasting plasma glucose and (**B**) high body-mass index by sex from 1990 to 2030

The ASMR of pancreatic cancer attributable to high FPG will increase from 0.72 in 2019 to 0.85 by 2030 in males, and from 0.51 to 0.64 in females. The ASMR of pancreatic cancer attributable to high BMI will increase from 0.36 in 2019 to 0.42 by 2030 in males, and from 0.42 to 0.48 in females. In the next 10 years, the ASMR of pancreatic cancer attributable to high FPG will still be higher in males than in females, and the ASMR of pancreatic cancer attributable to high BMI will be higher in females than in males (Fig. 5).

We also predicted ASMRs of pancreatic cancer attributable to high FPG and high BMI in seven GBD super-regions (Figure S9). The results showed that the ASMR of pancreatic cancer attributable to high FPG will continue to increase in the next 10 years, while the female ASMR of pancreatic cancer attributable to high BMI may slightly decline in high-income region, Southeast Asia, East Asia, and Oceania, and South Asia.

## Discussion

The global burden of pancreatic cancer has doubled in the past 20 years [3]. Metabolic disorders are known to affect pancreatic cancer progression and development [9, 23]. Understanding the burden of pancreatic cancer attributable to metabolic risks worldwide and implementing early detection and management are important steps toward cancer prevention. Our study showed that pancreatic cancer attributable to metabolic risk factors, including high BMI and high FPG, caused high mortality and DALYs, especially in high-income countries, and that the ASMR will continue to increase in the future. Metabolic factors are still responsible for the high global burden of pancreatic cancer. Obesity and diabetes should be further controlled to reduce metabolic risk-related pancreatic cancer.

Our research indicates that pancreatic cancer appears to occur primarily in high-income regions. The high burden of pancreatic cancer in high-income countries may be due to increased lifestyle-related metabolic risks; metabolic risks are more prevalent in developed countries [10]. One predictive study demonstrated that pancreatic cancer is projected to become the second leading cause of cancer-related death by 2030 in the USA [24]. By 2025, pancreatic cancer is predicted to cause more than one hundred thousand deaths in Europe [25]. Additionally, pancreatic cancer is typically a disease of older people. Age is one of the most important risks for pancreatic cancer [26]. As the survival for pancreatic cancer improves, the number of pancreatic cancer deaths has increased [10]. The global age-standardized prevalence rate of type 2 diabetes has increased nearly 50% during the past 30 years; and the prevalence of age-standardized obesity has increased nearly three times in men and more than two times in women [27, 28]. Therefore, diabetes and high BMI are expected to further contribute to the burden of pancreatic cancer.

A study has shown that every 0.56 mmol/L rise in FPG is linked to a 14% increase in pancreatic cancer occurrence [29]. The higher burden of pancreatic cancer may be due to the elevated occurrence of diabetes in some regions, as well as the lack of effective methods for managing diabetes-related cancer patients. High-income regions, such as the United States and Western Europe, have a high prevalence of diabetes despite public health measures [30]. The consumption of snacks, fast food, and beverages has increased significantly in some developing countries in recent years, leading to a higher prevalence of diabetes [31].

High BMI is another essential risk for pancreatic cancer. Data from the Health Professional follow-up study and Nurse’s Health study showed that individuals with BMI > 30 kg/m^2^ are more likely to suffer pancreatic cancer than those with BMI < 23 kg/m^2^ (relative risk = 1.72, 95% CI: 1.19 to 2.4) [32]. The prevalence of high BMI showed a distribution pattern that matched socioeconomic development: developed regions, such as Europe, high-income North America and Australasia, had a higher prevalence of overweight and obesity [33]. During the past few years, European countries have exceedingly increased attributable disease burden via meat imports, while South Asian and African countries had notably low attributable death and DALY rates regarding meat trade [34]. However, high-income Asia Pacific countries showed a lower disease burden. In Singapore, health policy makers developed health-promoting public guidelines and encouraged the shaping of healthier habits among young people to prevent obesity [35].

Eating habits and physical activity levels are major factors affecting diabetes and obesity, and may contribute to metabolic disorders. During the past few decades, people’s dietary patterns have obviously changed, with a relevant increase in the consumption of foods rich in fat and sugar [36]. People currently live in economically and socially better circumstances. People are increasingly using high-tech products, cars and other modes of travel, and mental activity replaces physical activity, leading to sedentary behavior, which can also increase weight [37]. Moreover, as our pace of life increases rapidly, social pressure can further promote excessive food consumption and sedentary behavior. From 1980 to 2013, the proportion of people with high BMI increased to more than 30% in both sexes [33]. Early-onset type 2 diabetes is also a growing global health problem among adolescents and young adults [38]. Thus, more effective interventions and approaches are needed to reduce the future cancer burden attributable to metabolic disorders.

The burden of pancreatic cancer attributable to metabolic factors varies in different SDI quintiles. Since 1990, the regional ASMR of pancreatic cancer attributable to high BMI and FPG has generally increased. The distribution of disease burden was parallel to that of high BMI and FPG exposure among the different regions. It has been reported that the prevalence of obesity and diabetes is higher in high-income countries than in low-income countries [33, 39]. Differences between different SDI quintiles may also be influenced by the high detection rates of pancreatic cancer in high-income countries, with complete data coverage, cancer registry systems and rich datasets [10]. We also found that the burden of pancreatic cancer increased rapidly in several low-SDI and low-middle-SDI regions, such as South Asia, Central Asia and Western Sub-Saharan Africa. Inadequate health care and cancer prevention in these regions may contribute to the high mortality of pancreatic cancer. Moreover, our decomposition analysis suggested that in high or high-middle SDI regions, population aging and population growth have greatly affected the DALY of pancreatic cancer attributable to metabolic factors. With the further increase in the world population in the future and the aggravation of population aging, some preventive measures for pancreatic cancer are needed. We should focus on differences in medical care levels between regions, including health education and publicity, cancer registries, early cancer screening and cancer therapy.

This study has several limitations. First, a lack of detailed and high-quality data in low-income countries may lead to some information bias in registration databases. The reported epidemiological data are higher in developed regions mainly due to the high standard of health care, including endoscopic ultrasonography and PET-CT, while low-income countries lack effective early diagnostic methods for providing rich and available records. Second, due to the modeling methods used in GBD estimation, especially at the national level, the data obtained from GBD rely on modeling data due to the lack of raw data. Future studies should expand more rich data on pancreatic cancer and disaggregate common risk factors and life patterns according to age, sex, and ethnicity, especially in countries with high mortality and DALYs. The associations of socioeconomic factors with pancreatic cancer burden should also be studied further.

## Conclusion

Our study provides a comprehensive time trend analysis for the global burden of pancreatic cancer attributable to metabolic factors. Although the disease burden of pancreatic cancer is partly related to population growth and aging, metabolic factors are also important factors that cannot be ignored. It is essential to develop future strategies to reduce the prevalence of high BMI and diabetes to decrease the burden of pancreatic cancer due to metabolic factors.

### Electronic supplementary material

Below is the link to the electronic supplementary material.

Supplementary Material 1

Supplementary Material 2

## Data Availability

The data analyzed in this study were obtained from the Global Health Data Exchange at https://ghdx.healthdata.org.

## Abbreviations

ASR: Age-standardized rate
ASMR: Age-standardized mortality rates
BAPC: Bayes age-period-cohort
BMI: Body mass index
DALY: Disability-adjusted life-year
FPG: Fasting plasma glucose
GBD: Global Burden of Diseases
HAQ: Healthcare access and quality
UIs: Uncertainty intervals
SDI: Socio-demographic index
