# Supplementary material for: Global burden of pancreatic cancer attributable to metabolic risks from 1990 to 2019, with projections of mortality to 2030
Source: BMC Public Health. 2024 Feb 13;24:456. doi: 10.1186/s12889-024-17875-6 (PMC10865635; doi:10.1186/s12889-024-17875-6)
Supplement: Supplementary file 1 — Supplementary Material 1 [file 12889_2024_17875_MOESM1_ESM.docx]

**Supplementary file 1** Supplementary figures

**
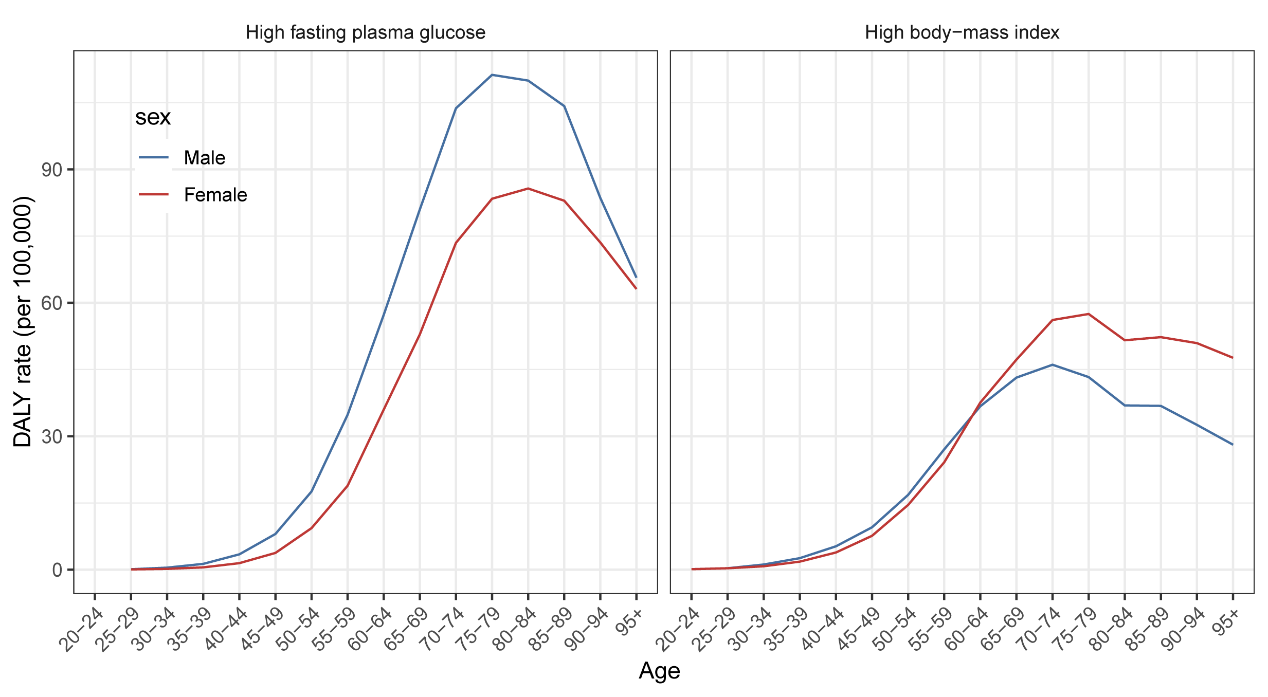
**

**Figure S1** The global age-specific DALY rates of pancreatic cancer attributable to high fasting plasma glucose and high body-mass index by sex in 2019. DALY: disability-adjusted life year

**
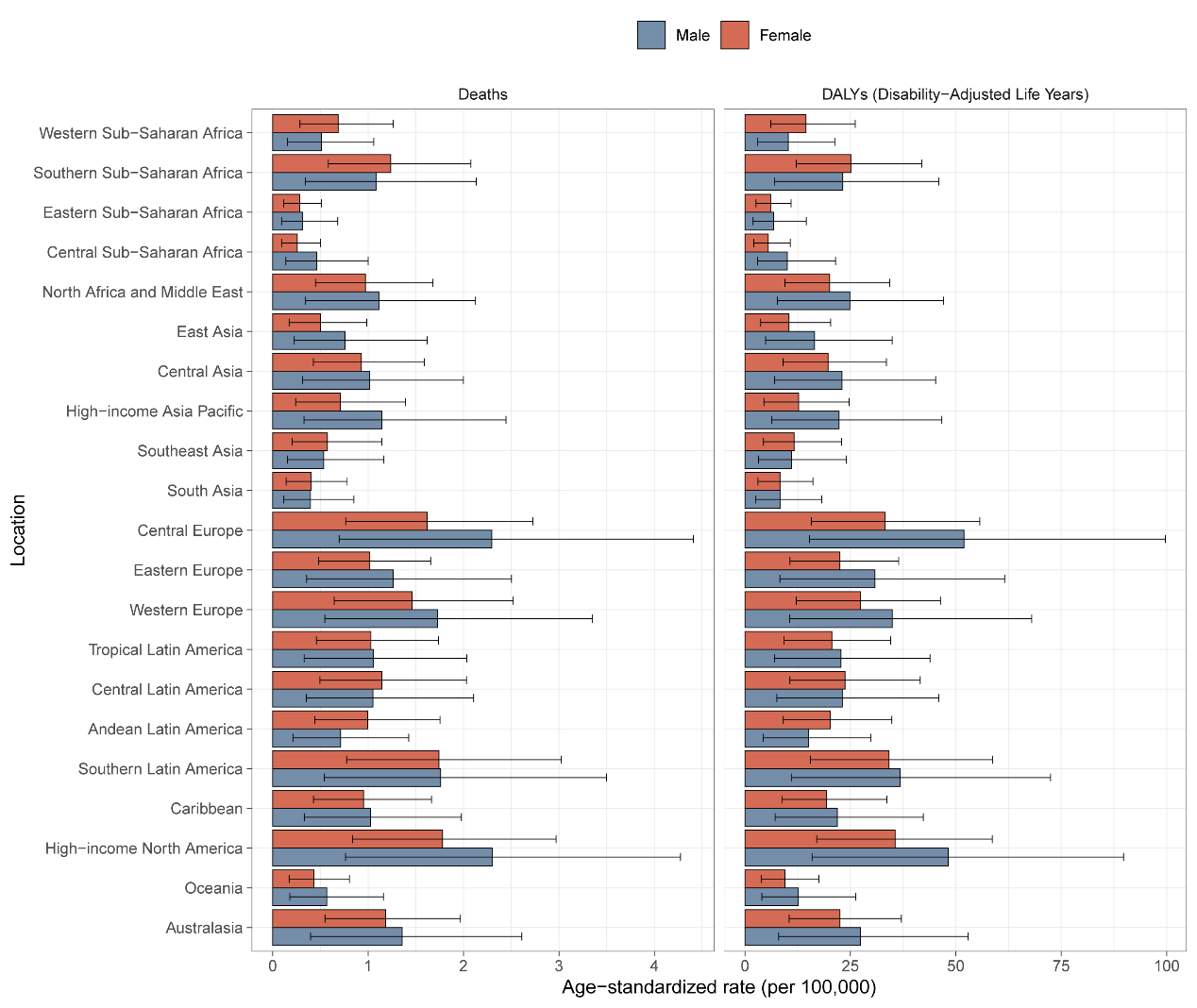
Figure S2** The age-standardized mortality and DALY rates of pancreatic cancer attributable to high fasting plasma glucose and high body-mass index in 21 GBD regions by sex, 2019

**
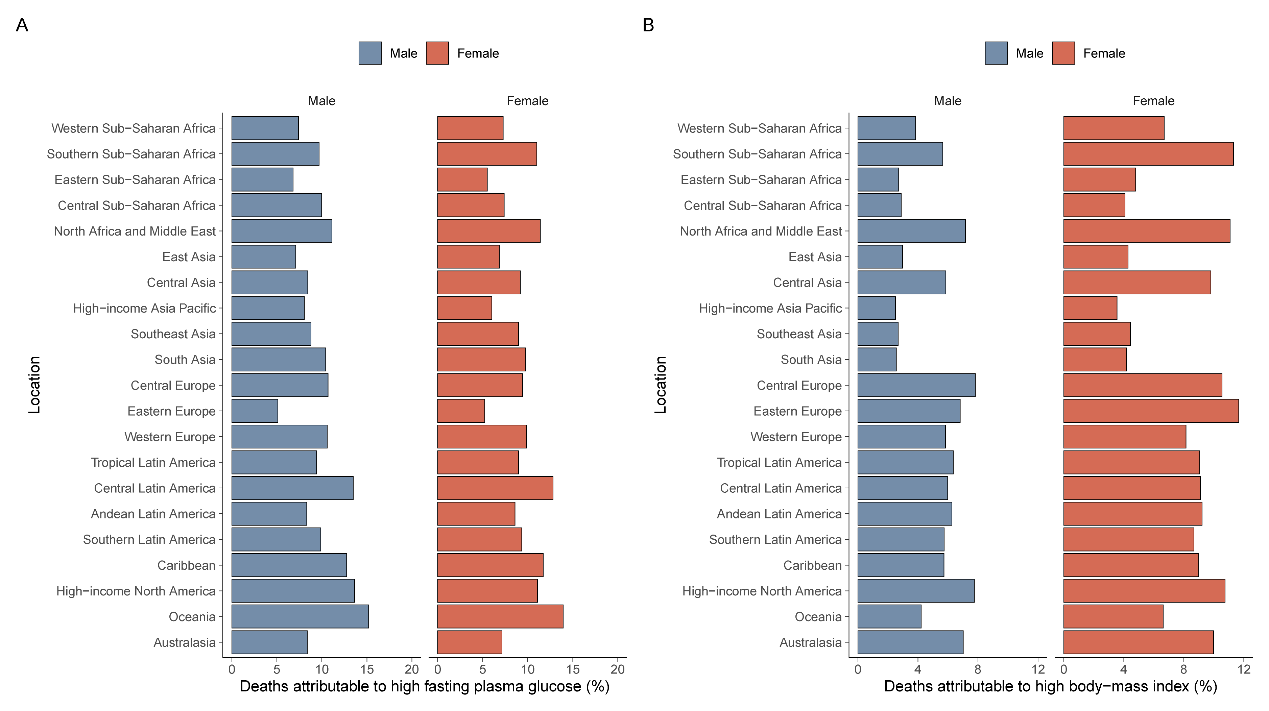
Figure S3** Fraction of pancreatic cancer age-specific mortality attributable to metabolic risks by age groups for male and female in 2019

**
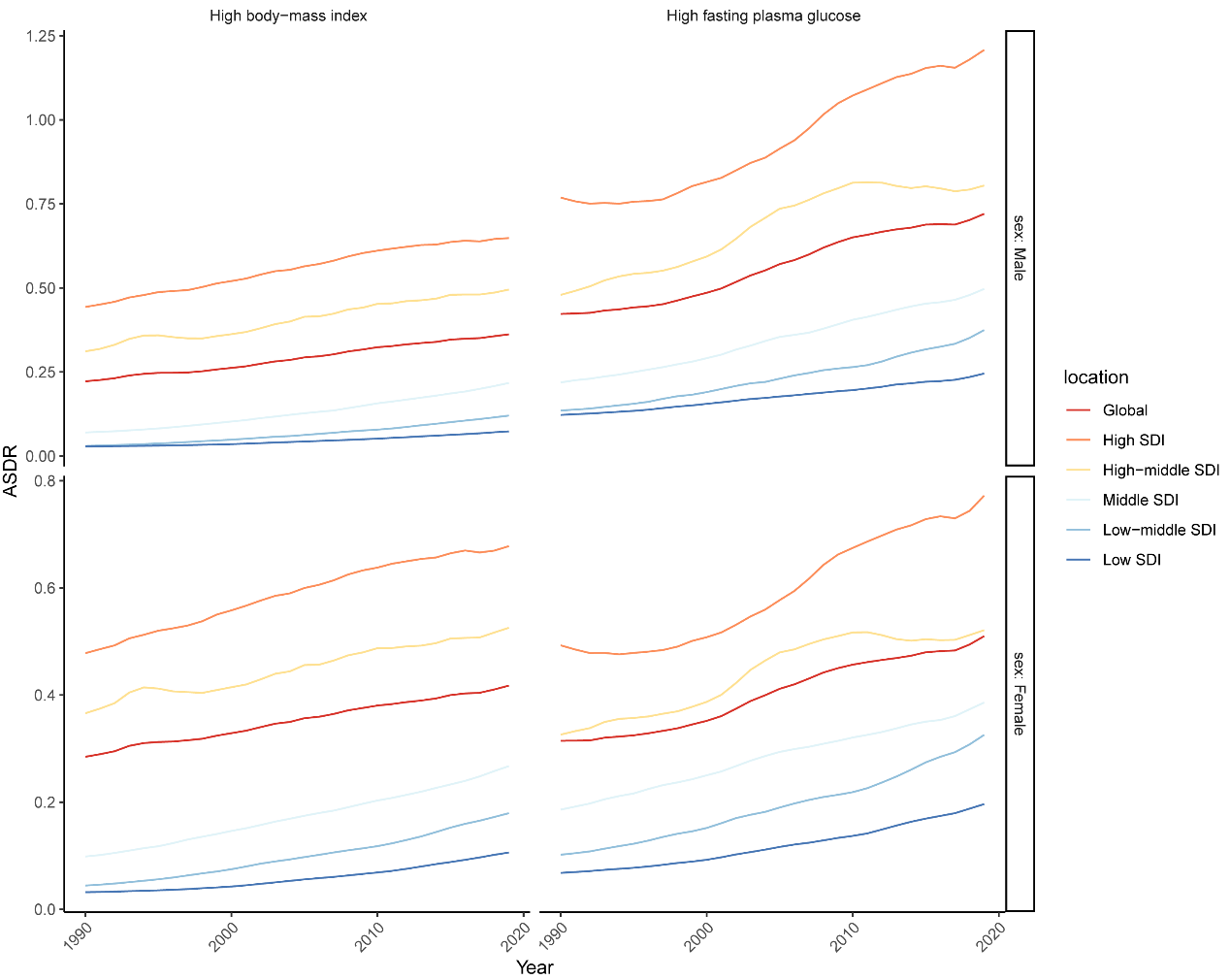
Figure S4** The trends in age-standardized mortality rate of metabolic risk-related pancreatic cancer in different SDI regions, from 1990 to 2019.

**
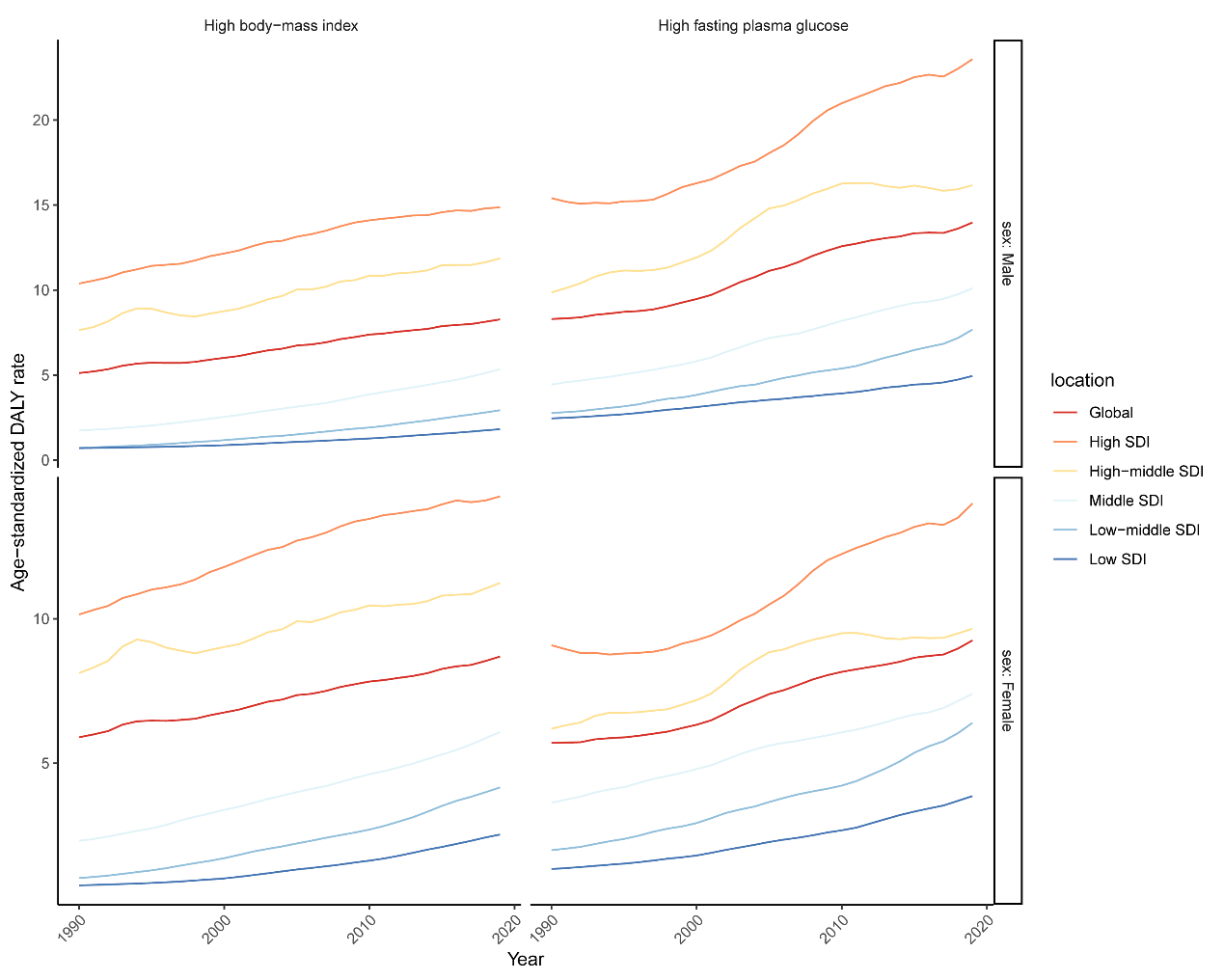
Figure S5** The trends in age-standardized DALY rate of metabolic risk-related pancreatic cancer in different SDI regions, from 1990 to 2019.

**
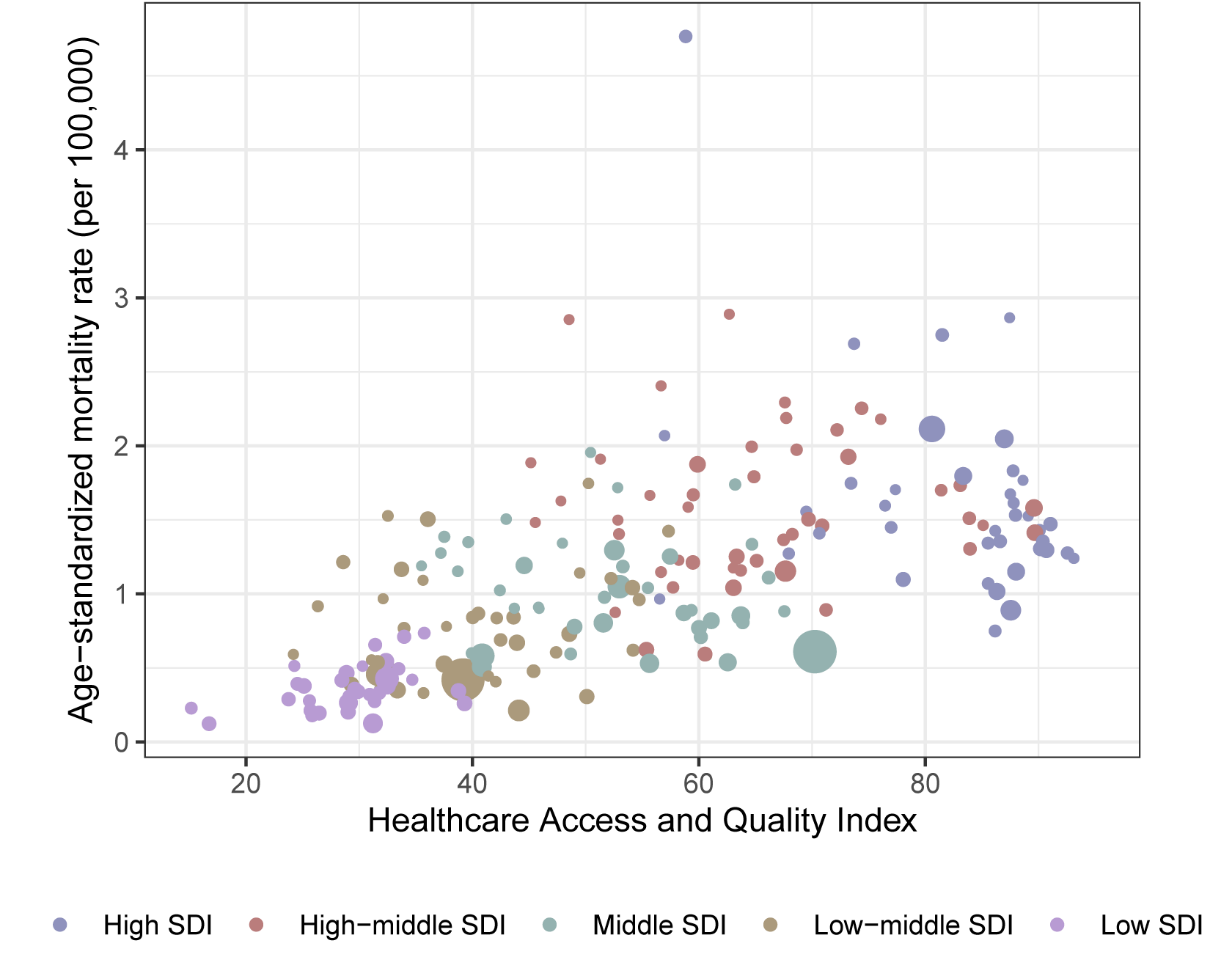
Figure S6** Association between age-standardized mortality rate and healthcare access and quality index in 2019. DALY: disability-adjusted life year

**
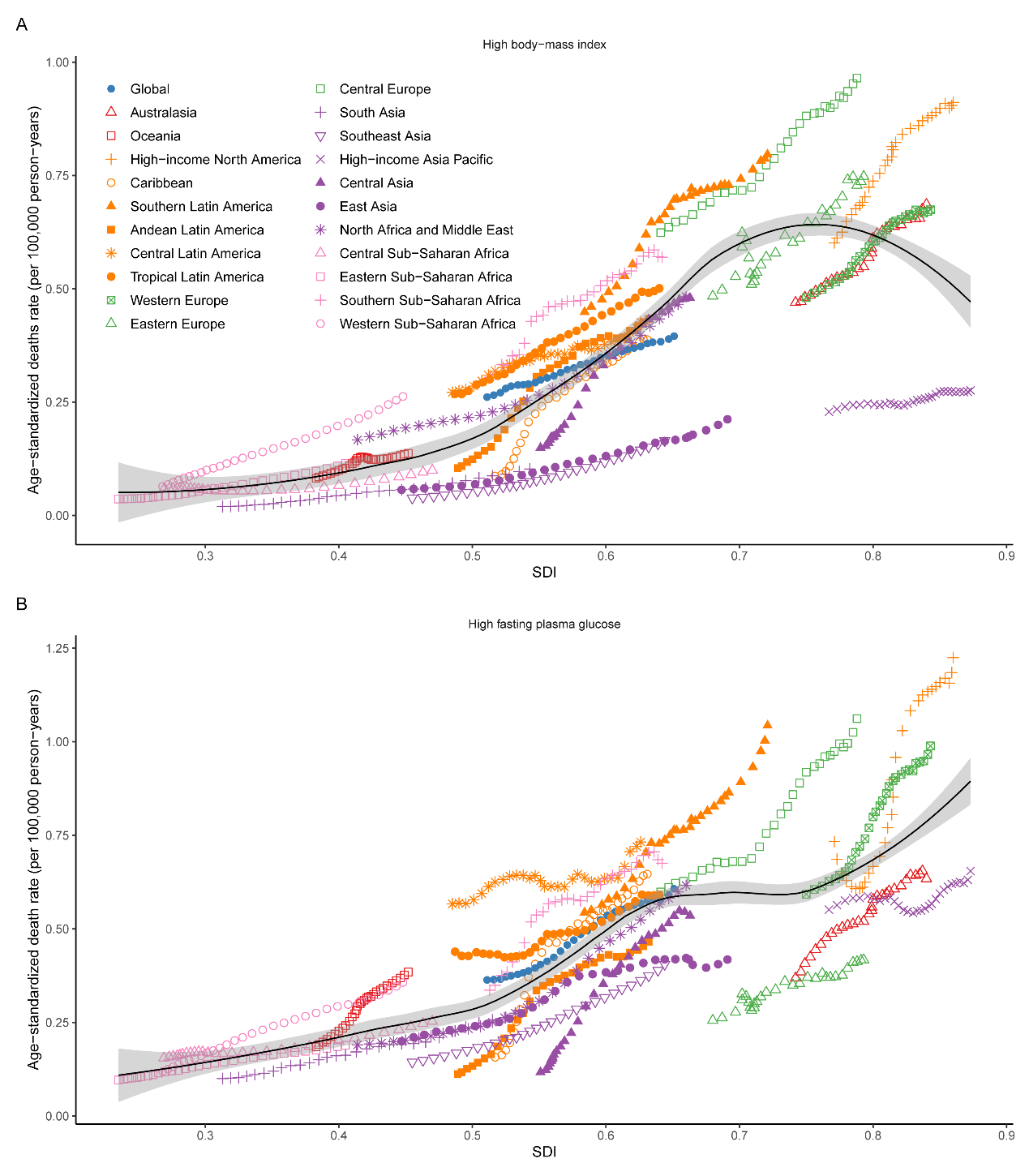
Figure S7** The relationship between age-standardized mortality rate of pancreatic cancer attributable to **(A)** high body-mass index and **(B)** high fasting plasma glucose and SDI among 21 GBD regions, 1990 to 2019

**
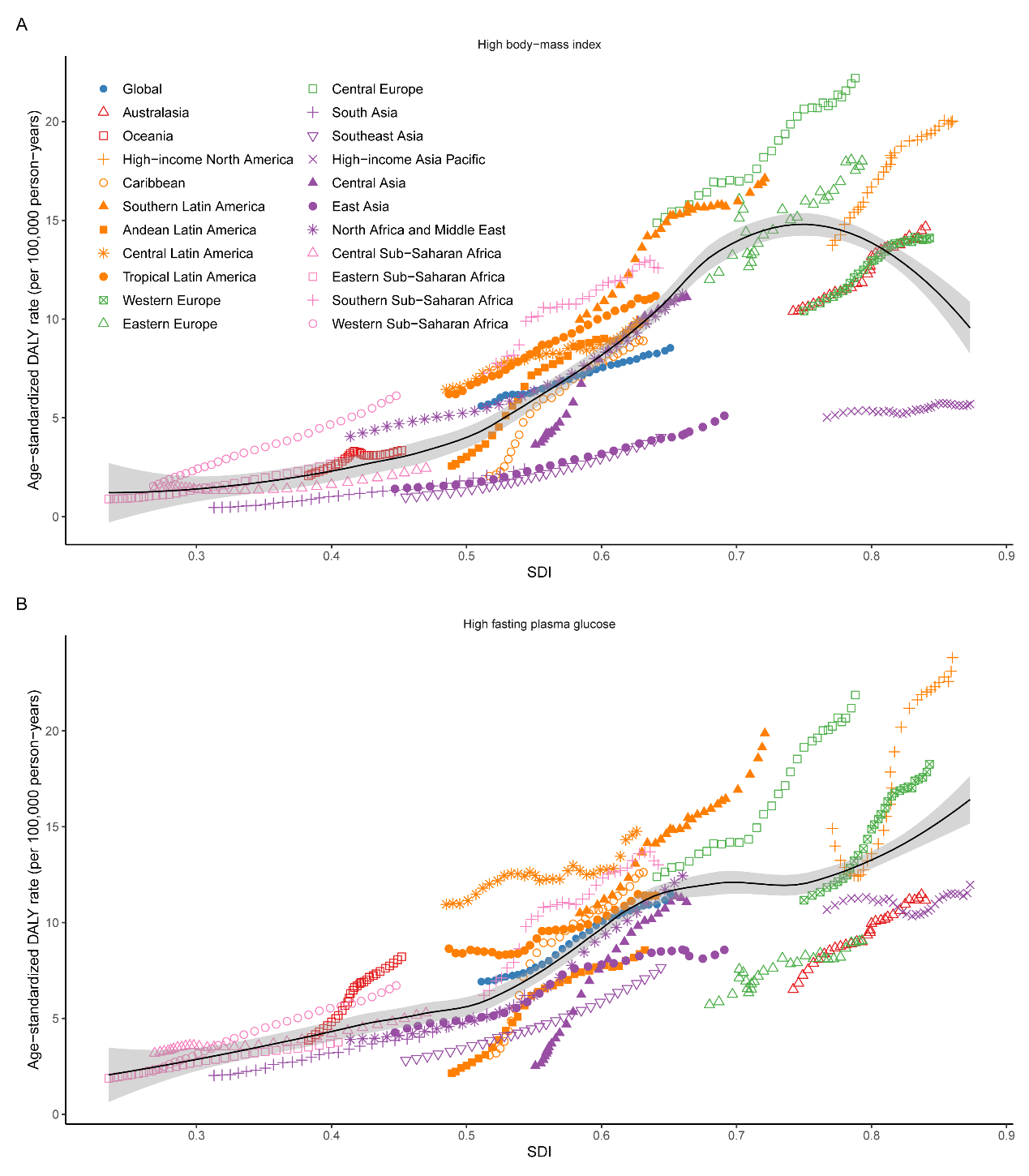
Figure S8** The relationship between age-standardized DALY rate of pancreatic cancer attributable to **(A)** high body-mass index and **(B)** high fasting plasma glucose and SDI among 21 GBD regions, 1990 to 2019

**
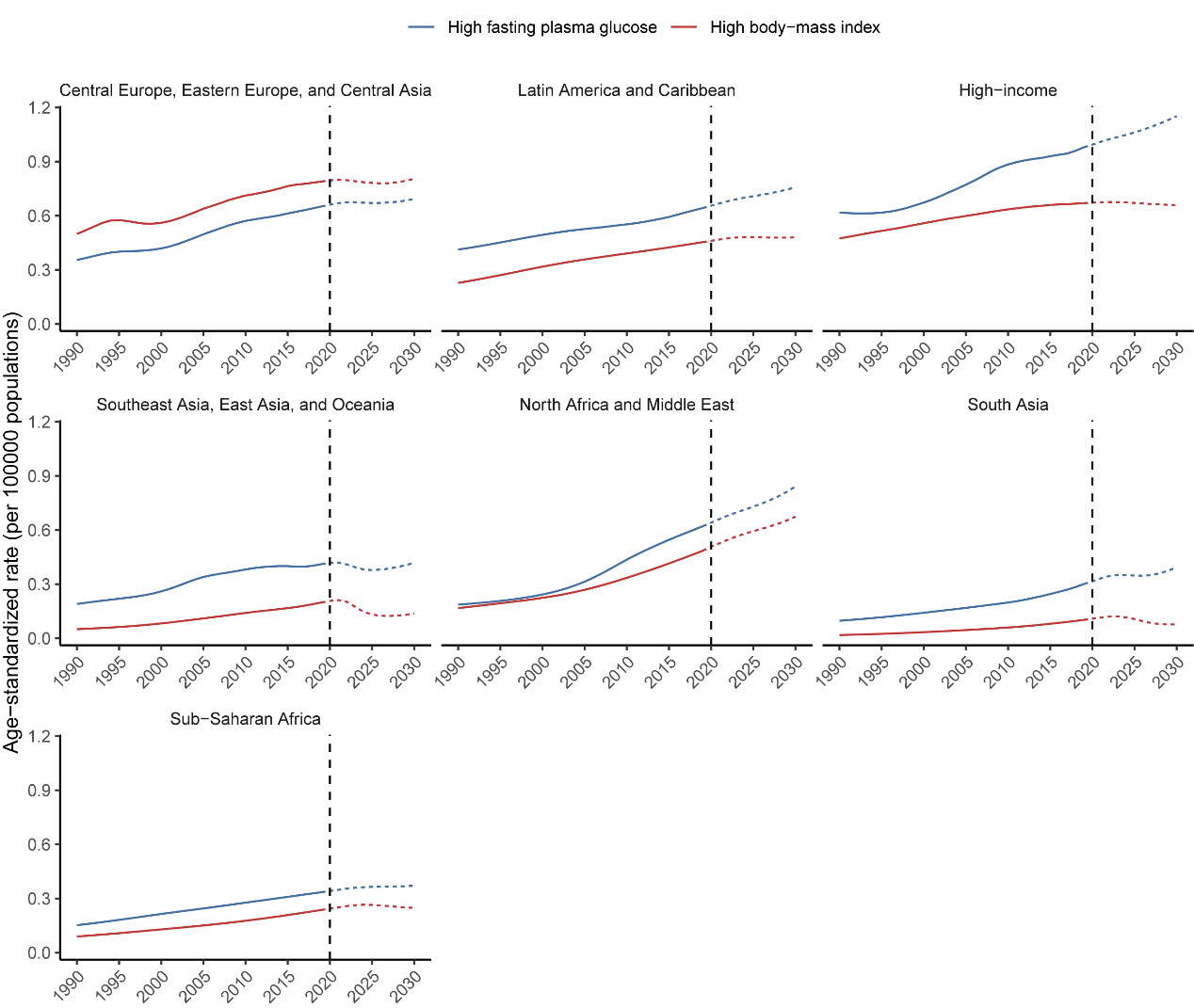
Figure S9** Time trend of age-standardized mortality rate of pancreatic cancer attributable to high fasting plasma glucose and high body-mass index in seven GBD super-regions from 1990 to 2030
