# Supplementary material for: Global burden of pancreatic cancer attributable to metabolic risks from 1990 to 2019, with projections of mortality to 2030
Source: BMC Public Health. 2024 Feb 13;24:456. doi: 10.1186/s12889-024-17875-6 (PMC10865635; doi:10.1186/s12889-024-17875-6)
Supplement: Supplementary file 2 — Supplementary Material 2 [file 12889_2024_17875_MOESM2_ESM.docx]

**Supplementary file 2**: Supplementary tables

**Table S1** The deaths and age-standardized mortality rate of pancreatic cancer attributable to metabolic risk among in countries/territories, 1990 and 2019

| Country/region | Deaths in 1990 | ASMR in 1990 | Deaths in 2019 | ASMR in 2019 |
| --- | --- | --- | --- | --- |
| Afghanistan | 13 (4-33) | 0.2 (0.06-0.49) | 50 (18-109) | 0.46 (0.17-1.01) |
| Albania | 7 (3-13) | 0.35 (0.15-0.65) | 39 (16-71) | 0.88 (0.36-1.63) |
| Algeria | 30 (12-56) | 0.27 (0.11-0.52) | 265 (115-474) | 0.87 (0.38-1.56) |
| American Samoa | 0 (0-0) | 0.74 (0.32-1.33) | 1 (0-1) | 1.48 (0.66-2.57) |
| Andorra | 1 (0-1) | 1.05 (0.4-2.02) | 2 (1-4) | 1.53 (0.62-2.78) |
| Angola | 6 (1-13) | 0.17 (0.04-0.38) | 37 (14-75) | 0.39 (0.14-0.76) |
| Antigua and Barbuda | 0 (0-0) | 0.19 (0.07-0.37) | 1 (0-2) | 1.23 (0.5-2.25) |
| Argentina | 322 (129-585) | 1.01 (0.4-1.84) | 1032 (469-1822) | 1.88 (0.85-3.31) |
| Armenia | 18 (7-32) | 0.69 (0.29-1.26) | 72 (32-126) | 1.74 (0.78-3.03) |
| Australia | 164 (71-284) | 0.84 (0.37-1.45) | 570 (265-974) | 1.31 (0.6-2.22) |
| Austria | 129 (56-229) | 1.05 (0.45-1.87) | 287 (126-513) | 1.53 (0.67-2.71) |
| Azerbaijan | 16 (7-29) | 0.34 (0.15-0.61) | 100 (44-174) | 1.18 (0.53-2.1) |
| Bahamas | 0 (0-1) | 0.28 (0.12-0.49) | 3 (1-6) | 0.87 (0.37-1.62) |
| Bahrain | 2 (1-4) | 1.44 (0.63-2.57) | 16 (7-29) | 2.29 (1-4.09) |
| Bangladesh | 35 (7-90) | 0.08 (0.02-0.22) | 260 (75-629) | 0.21 (0.06-0.52) |
| Barbados | 1 (0-1) | 0.23 (0.1-0.42) | 8 (4-14) | 1.59 (0.7-2.86) |
| Belarus | 65 (29-113) | 0.5 (0.22-0.87) | 143 (62-252) | 0.89 (0.38-1.58) |
| Belgium | 157 (67-284) | 0.99 (0.42-1.79) | 329 (144-590) | 1.36 (0.59-2.44) |
| Belize | 0 (0-0) | 0.19 (0.08-0.35) | 3 (1-5) | 1.14 (0.52-2.04) |
| Benin | 3 (1-7) | 0.18 (0.06-0.36) | 28 (12-53) | 0.66 (0.26-1.24) |
| Bermuda | 0 (0-0) | 0.43 (0.19-0.73) | 2 (1-4) | 1.7 (0.74-3.06) |
| Bhutan | 0 (0-1) | 0.1 (0.02-0.26) | 2 (1-5) | 0.41 (0.13-0.93) |
| Bolivia (Plurinational State of) | 8 (3-16) | 0.27 (0.1-0.52) | 72 (29-141) | 0.87 (0.34-1.7) |
| Bosnia and Herzegovina | 35 (15-63) | 0.93 (0.39-1.68) | 120 (52-225) | 1.97 (0.86-3.68) |
| Botswana | 2 (1-4) | 0.37 (0.13-0.74) | 16 (7-29) | 1.39 (0.62-2.54) |
| Brazil | 563 (235-1018) | 0.7 (0.29-1.27) | 2421 (1093-4166) | 1.05 (0.47-1.81) |
| Brunei Darussalam | 1 (0-2) | 1.3 (0.39-2.53) | 5 (2-9) | 2.07 (0.74-3.83) |
| Bulgaria | 116 (50-199) | 0.92 (0.39-1.57) | 261 (114-472) | 1.79 (0.78-3.24) |
| Burkina Faso | 5 (1-11) | 0.13 (0.04-0.28) | 33 (13-66) | 0.42 (0.16-0.84) |
| Burundi | 3 (1-7) | 0.14 (0.04-0.3) | 7 (2-16) | 0.18 (0.06-0.4) |
| Cabo Verde | 0 (0-0) | 0.1 (0.04-0.2) | 7 (3-13) | 1.75 (0.71-3.31) |
| Cambodia | 5 (1-11) | 0.12 (0.03-0.27) | 48 (17-95) | 0.45 (0.16-0.9) |
| Cameroon | 15 (6-28) | 0.38 (0.15-0.7) | 121 (49-228) | 1.17 (0.47-2.22) |
| Canada | 294 (131-513) | 0.9 (0.4-1.57) | 927 (428-1578) | 1.3 (0.59-2.2) |
| Central African Republic | 2 (0-4) | 0.17 (0.05-0.39) | 4 (1-10) | 0.23 (0.07-0.52) |
| Chad | 2 (1-5) | 0.09 (0.03-0.19) | 14 (5-28) | 0.29 (0.1-0.59) |
| Chile | 85 (37-149) | 0.9 (0.39-1.57) | 353 (157-623) | 1.46 (0.65-2.57) |
| China | 1953 (579-4094) | 0.25 (0.07-0.53) | 11992 (4439-23315) | 0.61 (0.22-1.19) |
| Colombia | 125 (50-228) | 0.79 (0.31-1.45) | 434 (180-837) | 0.82 (0.34-1.58) |
| Comoros | 0 (0-1) | 0.19 (0.06-0.39) | 2 (1-3) | 0.35 (0.14-0.66) |
| Congo | 4 (1-8) | 0.38 (0.13-0.81) | 18 (7-36) | 0.77 (0.29-1.54) |
| Cook Islands | 0 (0-0) | 0.63 (0.25-1.17) | 0 (0-1) | 1.17 (0.51-2.1) |
| Costa Rica | 8 (3-14) | 0.47 (0.19-0.85) | 68 (29-127) | 1.34 (0.57-2.5) |
| Croatia | 74 (33-127) | 1.17 (0.52-2.02) | 156 (70-278) | 1.7 (0.75-3.05) |
| Cuba | 25 (10-46) | 0.24 (0.1-0.45) | 215 (94-383) | 1.11 (0.49-1.97) |
| Cyprus | 5 (2-10) | 0.67 (0.25-1.27) | 28 (11-51) | 1.43 (0.57-2.62) |
| Czechia | 262 (115-454) | 1.88 (0.83-3.26) | 602 (272-1067) | 2.75 (1.24-4.89) |
| Côte d'Ivoire | 8 (3-15) | 0.23 (0.08-0.46) | 61 (25-116) | 0.68 (0.27-1.32) |
| Democratic People's Republic of Korea | 25 (7-56) | 0.17 (0.05-0.37) | 97 (28-207) | 0.31 (0.09-0.65) |
| Democratic Republic of the Congo | 27 (9-57) | 0.2 (0.07-0.43) | 83 (29-167) | 0.27 (0.09-0.54) |
| Denmark | 54 (23-97) | 0.65 (0.28-1.15) | 162 (70-292) | 1.34 (0.59-2.4) |
| Djibouti | 0 (0-0) | 0.15 (0.04-0.32) | 2 (1-4) | 0.41 (0.14-0.86) |
| Dominica | 0 (0-0) | 0.28 (0.12-0.5) | 2 (1-3) | 1.89 (0.81-3.42) |
| Dominican Republic | 4 (2-8) | 0.12 (0.04-0.22) | 43 (17-85) | 0.48 (0.18-0.94) |
| Ecuador | 12 (5-20) | 0.23 (0.1-0.41) | 154 (68-275) | 1.07 (0.47-1.92) |
| Egypt | 60 (25-107) | 0.21 (0.09-0.38) | 496 (205-901) | 0.8 (0.33-1.46) |
| El Salvador | 6 (2-10) | 0.2 (0.08-0.37) | 57 (23-109) | 0.96 (0.39-1.82) |
| Equatorial Guinea | 0 (0-1) | 0.15 (0.04-0.38) | 4 (2-8) | 1.02 (0.38-1.98) |
| Eritrea | 1 (0-2) | 0.1 (0.03-0.22) | 6 (2-13) | 0.28 (0.1-0.58) |
| Estonia | 20 (9-33) | 0.95 (0.44-1.61) | 44 (21-75) | 1.6 (0.74-2.72) |
| Eswatini | 2 (1-3) | 0.66 (0.29-1.25) | 8 (3-15) | 1.53 (0.64-2.89) |
| Ethiopia | 13 (3-35) | 0.08 (0.02-0.2) | 45 (15-102) | 0.13 (0.04-0.29) |
| Fiji | 2 (1-3) | 0.5 (0.2-0.98) | 8 (3-14) | 1.15 (0.48-2.11) |
| Finland | 101 (44-179) | 1.38 (0.6-2.45) | 241 (104-435) | 1.83 (0.79-3.31) |
| France | 572 (244-1021) | 0.67 (0.28-1.2) | 1684 (736-2986) | 1.15 (0.5-2.03) |
| Gabon | 2 (1-5) | 0.43 (0.15-0.85) | 13 (5-26) | 1.35 (0.55-2.65) |
| Gambia | 0 (0-1) | 0.11 (0.04-0.24) | 4 (1-7) | 0.42 (0.16-0.85) |
| Georgia | 27 (11-48) | 0.44 (0.19-0.78) | 63 (27-113) | 1.04 (0.45-1.89) |
| Germany | 1579 (684-2826) | 1.2 (0.52-2.14) | 4180 (1790-7401) | 2.05 (0.89-3.63) |
| Ghana | 20 (7-41) | 0.37 (0.13-0.76) | 220 (96-400) | 1.5 (0.65-2.75) |
| Greece | 164 (69-295) | 1.06 (0.45-1.91) | 381 (167-676) | 1.51 (0.66-2.66) |
| Greenland | 0 (0-1) | 1.56 (0.68-2.66) | 2 (1-3) | 2.89 (1.28-5.2) |
| Grenada | 0 (0-0) | 0.24 (0.09-0.46) | 2 (1-4) | 1.96 (0.8-3.55) |
| Guam | 0 (0-1) | 0.62 (0.26-1.14) | 2 (1-3) | 0.97 (0.42-1.72) |
| Guatemala | 5 (2-10) | 0.17 (0.06-0.33) | 88 (34-175) | 0.84 (0.32-1.67) |
| Guinea | 3 (1-5) | 0.08 (0.03-0.17) | 11 (4-20) | 0.21 (0.08-0.41) |
| Guinea-Bissau | 1 (0-1) | 0.18 (0.05-0.41) | 3 (1-6) | 0.51 (0.18-1.04) |
| Guyana | 1 (0-2) | 0.27 (0.1-0.51) | 8 (3-14) | 1.28 (0.51-2.39) |
| Haiti | 4 (1-8) | 0.13 (0.04-0.28) | 24 (8-54) | 0.39 (0.12-0.86) |
| Honduras | 5 (2-10) | 0.27 (0.1-0.51) | 46 (16-100) | 0.84 (0.28-1.8) |
| Hungary | 214 (96-358) | 1.45 (0.65-2.43) | 446 (205-792) | 2.25 (1.03-3.98) |
| Iceland | 3 (1-5) | 0.97 (0.42-1.7) | 7 (3-13) | 1.24 (0.54-2.2) |
| India | 454 (138-996) | 0.12 (0.04-0.27) | 4446 (1615-8629) | 0.42 (0.15-0.81) |
| Indonesia | 120 (37-250) | 0.14 (0.04-0.29) | 1123 (377-2411) | 0.58 (0.19-1.28) |
| Iran (Islamic Republic of) | 53 (22-105) | 0.23 (0.09-0.46) | 574 (257-1000) | 0.85 (0.38-1.49) |
| Iraq | 36 (14-70) | 0.49 (0.19-0.97) | 262 (112-472) | 1.25 (0.54-2.26) |
| Ireland | 36 (16-65) | 0.87 (0.38-1.57) | 110 (49-196) | 1.43 (0.63-2.54) |
| Israel | 57 (25-101) | 1.16 (0.5-2.06) | 208 (93-372) | 1.73 (0.78-3.08) |
| Italy | 1038 (442-1865) | 1.14 (0.48-2.04) | 2446 (1044-4402) | 1.58 (0.69-2.84) |
| Jamaica | 3 (1-6) | 0.19 (0.08-0.34) | 31 (13-57) | 1.04 (0.44-1.93) |
| Japan | 1300 (444-2569) | 0.77 (0.27-1.53) | 3583 (1210-7180) | 0.89 (0.31-1.78) |
| Jordan | 5 (2-9) | 0.45 (0.19-0.81) | 70 (33-119) | 1.22 (0.57-2.1) |
| Kazakhstan | 20 (9-34) | 0.17 (0.08-0.28) | 203 (94-347) | 1.21 (0.57-2.08) |
| Kenya | 8 (2-17) | 0.1 (0.03-0.23) | 71 (30-133) | 0.35 (0.14-0.67) |
| Kiribati | 0 (0-0) | 0.33 (0.13-0.65) | 0 (0-1) | 0.59 (0.23-1.15) |
| Kuwait | 4 (2-7) | 0.79 (0.36-1.41) | 30 (14-53) | 1.45 (0.65-2.55) |
| Kyrgyzstan | 10 (4-17) | 0.33 (0.15-0.58) | 27 (12-48) | 0.62 (0.27-1.11) |
| Lao People's Democratic Republic | 3 (1-8) | 0.18 (0.05-0.42) | 18 (7-36) | 0.49 (0.17-0.96) |
| Latvia | 33 (15-55) | 0.9 (0.41-1.53) | 64 (30-109) | 1.56 (0.7-2.65) |
| Lebanon | 10 (4-18) | 0.47 (0.19-0.87) | 73 (32-128) | 1.4 (0.62-2.47) |
| Lesotho | 2 (1-5) | 0.25 (0.09-0.51) | 10 (4-19) | 0.92 (0.37-1.72) |
| Liberia | 3 (1-6) | 0.26 (0.1-0.53) | 13 (5-25) | 0.74 (0.29-1.42) |
| Libya | 10 (4-21) | 0.59 (0.22-1.21) | 78 (33-144) | 1.67 (0.7-3.11) |
| Lithuania | 36 (16-62) | 0.8 (0.35-1.39) | 75 (35-128) | 1.27 (0.58-2.19) |
| Luxembourg | 5 (2-9) | 0.94 (0.41-1.65) | 17 (7-32) | 1.67 (0.7-3.04) |
| Madagascar | 5 (2-11) | 0.12 (0.04-0.24) | 19 (7-38) | 0.2 (0.08-0.42) |
| Malawi | 5 (1-10) | 0.15 (0.04-0.31) | 22 (8-42) | 0.34 (0.13-0.66) |
| Malaysia | 17 (6-35) | 0.21 (0.07-0.43) | 151 (61-286) | 0.62 (0.24-1.18) |
| Maldives | 0 (0-0) | 0.24 (0.06-0.53) | 1 (1-3) | 0.58 (0.21-1.13) |
| Mali | 5 (2-10) | 0.14 (0.04-0.28) | 27 (10-55) | 0.36 (0.13-0.72) |
| Malta | 5 (2-9) | 1.11 (0.44-2.07) | 14 (6-27) | 1.46 (0.61-2.71) |
| Marshall Islands | 0 (0-0) | 0.41 (0.14-0.83) | 0 (0-1) | 0.97 (0.34-1.89) |
| Mauritania | 3 (1-5) | 0.29 (0.11-0.56) | 16 (7-28) | 0.84 (0.36-1.48) |
| Mauritius | 4 (1-7) | 0.53 (0.2-1.01) | 20 (8-38) | 1.15 (0.46-2.19) |
| Mexico | 437 (187-763) | 1.12 (0.47-1.96) | 1468 (657-2587) | 1.3 (0.58-2.28) |
| Micronesia (Federated States of) | 0 (0-0) | 0.42 (0.17-0.8) | 1 (0-1) | 1.09 (0.43-2.1) |
| Monaco | 1 (1-2) | 1.49 (0.66-2.67) | 3 (1-5) | 2.87 (1.23-5.03) |
| Mongolia | 2 (1-4) | 0.23 (0.1-0.42) | 13 (5-24) | 0.6 (0.25-1.09) |
| Montenegro | 7 (3-12) | 1.13 (0.51-1.97) | 22 (10-38) | 2.18 (0.98-3.84) |
| Morocco | 27 (10-52) | 0.21 (0.08-0.41) | 212 (84-400) | 0.73 (0.28-1.38) |
| Mozambique | 6 (2-13) | 0.12 (0.03-0.26) | 36 (13-73) | 0.38 (0.13-0.78) |
| Myanmar | 37 (10-87) | 0.18 (0.05-0.42) | 219 (77-431) | 0.53 (0.18-1.05) |
| Namibia | 1 (1-3) | 0.2 (0.08-0.4) | 8 (3-14) | 0.6 (0.25-1.1) |
| Nauru | 0 (0-0) | 0.63 (0.25-1.19) | 0 (0-0) | 1.19 (0.44-2.34) |
| Nepal | 5 (1-14) | 0.07 (0.02-0.18) | 70 (20-162) | 0.34 (0.1-0.81) |
| Netherlands | 195 (82-352) | 0.95 (0.4-1.72) | 527 (225-938) | 1.47 (0.63-2.6) |
| New Zealand | 29 (12-51) | 0.72 (0.31-1.28) | 87 (39-147) | 1.07 (0.48-1.82) |
| Nicaragua | 5 (2-9) | 0.33 (0.11-0.69) | 44 (18-78) | 1.1 (0.45-1.97) |
| Niger | 2 (1-3) | 0.06 (0.02-0.14) | 13 (4-28) | 0.19 (0.06-0.43) |
| Nigeria | 60 (21-126) | 0.16 (0.05-0.33) | 334 (138-613) | 0.46 (0.18-0.85) |
| Niue | 0 (0-0) | 0.69 (0.27-1.28) | 0 (0-0) | 1.63 (0.67-2.97) |
| North Macedonia | 17 (7-30) | 0.93 (0.41-1.67) | 70 (30-128) | 2.19 (0.95-3.98) |
| Northern Mariana Islands | 0 (0-0) | 0.62 (0.27-1.11) | 1 (0-1) | 1.67 (0.75-2.88) |
| Norway | 71 (28-131) | 0.99 (0.4-1.82) | 136 (57-246) | 1.34 (0.57-2.43) |
| Oman | 1 (0-3) | 0.25 (0.08-0.54) | 18 (8-31) | 1.37 (0.62-2.34) |
| Pakistan | 67 (20-144) | 0.13 (0.04-0.27) | 410 (150-804) | 0.43 (0.15-0.85) |
| Palau | 0 (0-0) | 1.46 (0.6-2.72) | 1 (0-1) | 2.85 (1.27-5.17) |
| Palestine | 4 (1-8) | 0.45 (0.16-0.97) | 29 (12-53) | 1.42 (0.59-2.59) |
| Panama | 4 (1-8) | 0.3 (0.1-0.59) | 37 (15-70) | 0.89 (0.36-1.71) |
| Papua New Guinea | 2 (1-5) | 0.14 (0.04-0.33) | 12 (4-26) | 0.27 (0.08-0.61) |
| Paraguay | 5 (2-9) | 0.25 (0.1-0.46) | 52 (21-97) | 0.98 (0.41-1.84) |
| Peru | 21 (8-38) | 0.19 (0.07-0.34) | 243 (106-448) | 0.77 (0.34-1.42) |
| Philippines | 76 (27-151) | 0.3 (0.1-0.6) | 354 (135-670) | 0.51 (0.19-0.97) |
| Poland | 588 (265-998) | 1.35 (0.61-2.3) | 1378 (615-2408) | 1.93 (0.86-3.37) |
| Portugal | 130 (52-239) | 0.93 (0.37-1.71) | 334 (140-610) | 1.3 (0.55-2.37) |
| Puerto Rico | 14 (6-25) | 0.4 (0.18-0.69) | 105 (46-196) | 1.41 (0.63-2.61) |
| Qatar | 1 (0-2) | 1.14 (0.45-2.16) | 14 (6-25) | 2.69 (1.2-4.7) |
| Republic of Korea | 188 (63-367) | 0.69 (0.23-1.34) | 904 (325-1726) | 1.02 (0.37-1.95) |
| Republic of Moldova | 32 (14-54) | 0.71 (0.31-1.23) | 68 (31-117) | 1.16 (0.52-2) |
| Romania | 202 (87-352) | 0.71 (0.31-1.24) | 562 (252-985) | 1.5 (0.66-2.64) |
| Russian Federation | 1437 (673-2440) | 0.79 (0.37-1.35) | 2746 (1291-4595) | 1.15 (0.54-1.93) |
| Rwanda | 5 (2-11) | 0.19 (0.06-0.41) | 17 (7-33) | 0.33 (0.13-0.63) |
| Saint Kitts and Nevis | 0 (0-0) | 0.34 (0.14-0.62) | 1 (0-2) | 1.91 (0.81-3.44) |
| Saint Lucia | 0 (0-1) | 0.35 (0.14-0.65) | 4 (2-7) | 1.72 (0.71-3.14) |
| Saint Vincent and the Grenadines | 0 (0-0) | 0.26 (0.1-0.49) | 2 (1-3) | 1.34 (0.55-2.45) |
| Samoa | 1 (0-1) | 0.68 (0.27-1.25) | 1 (1-2) | 0.9 (0.37-1.71) |
| San Marino | 0 (0-1) | 1.03 (0.43-1.86) | 1 (0-3) | 1.77 (0.67-3.63) |
| Sao Tome and Principe | 0 (0-0) | 0.13 (0.05-0.26) | 0 (0-1) | 0.44 (0.17-0.9) |
| Saudi Arabia | 14 (5-29) | 0.28 (0.1-0.57) | 190 (87-320) | 1.25 (0.58-2.11) |
| Senegal | 7 (3-14) | 0.25 (0.09-0.5) | 47 (19-88) | 0.71 (0.28-1.33) |
| Serbia | 110 (48-194) | 0.99 (0.44-1.74) | 348 (150-624) | 2.11 (0.91-3.79) |
| Seychelles | 0 (0-1) | 0.6 (0.23-1.16) | 2 (1-3) | 1.5 (0.57-2.77) |
| Sierra Leone | 2 (1-4) | 0.09 (0.03-0.2) | 10 (4-20) | 0.32 (0.12-0.62) |
| Singapore | 13 (4-25) | 0.63 (0.2-1.25) | 57 (22-108) | 0.75 (0.29-1.42) |
| Slovakia | 67 (29-116) | 1.1 (0.48-1.92) | 164 (75-290) | 1.75 (0.79-3.09) |
| Slovenia | 29 (13-52) | 1.17 (0.51-2.12) | 73 (32-130) | 1.61 (0.69-2.88) |
| Solomon Islands | 0 (0-1) | 0.19 (0.06-0.41) | 2 (1-3) | 0.51 (0.18-1.03) |
| Somalia | 2 (1-6) | 0.1 (0.03-0.26) | 7 (2-18) | 0.12 (0.03-0.33) |
| South Africa | 128 (56-232) | 0.67 (0.29-1.23) | 489 (235-830) | 1.19 (0.57-2.03) |
| South Sudan | 4 (1-8) | 0.19 (0.06-0.39) | 10 (4-21) | 0.31 (0.12-0.64) |
| Spain | 566 (242-1014) | 1.01 (0.43-1.81) | 1422 (644-2523) | 1.41 (0.65-2.49) |
| Sri Lanka | 18 (6-37) | 0.2 (0.07-0.39) | 144 (55-289) | 0.59 (0.23-1.19) |
| Sudan | 11 (4-27) | 0.13 (0.04-0.32) | 112 (41-249) | 0.67 (0.25-1.51) |
| Suriname | 1 (0-1) | 0.25 (0.1-0.46) | 9 (4-16) | 1.51 (0.62-2.74) |
| Sweden | 176 (76-316) | 1.11 (0.47-1.98) | 303 (131-528) | 1.36 (0.59-2.36) |
| Switzerland | 80 (34-145) | 0.74 (0.31-1.33) | 236 (102-427) | 1.28 (0.55-2.3) |
| Syrian Arab Republic | 11 (4-22) | 0.24 (0.09-0.47) | 80 (32-151) | 0.71 (0.29-1.33) |
| Tajikistan | 6 (2-15) | 0.25 (0.08-0.56) | 27 (10-52) | 0.69 (0.25-1.35) |
| Thailand | 79 (27-160) | 0.25 (0.08-0.51) | 539 (206-1080) | 0.54 (0.2-1.07) |
| Timor-Leste | 0 (0-1) | 0.1 (0.03-0.23) | 2 (1-5) | 0.33 (0.09-0.7) |
| Togo | 2 (1-3) | 0.15 (0.05-0.3) | 15 (6-30) | 0.49 (0.2-0.95) |
| Tokelau | 0 (0-0) | 0.36 (0.14-0.68) | 0 (0-0) | 0.9 (0.36-1.73) |
| Tonga | 0 (0-0) | 0.44 (0.17-0.85) | 1 (0-1) | 0.91 (0.35-1.72) |
| Trinidad and Tobago | 2 (1-4) | 0.28 (0.11-0.5) | 26 (11-49) | 1.4 (0.6-2.64) |
| Tunisia | 13 (5-25) | 0.28 (0.11-0.54) | 98 (41-185) | 0.81 (0.34-1.54) |
| Turkey | 230 (99-419) | 0.67 (0.29-1.22) | 1296 (580-2314) | 1.51 (0.68-2.69) |
| Turkmenistan | 3 (1-5) | 0.15 (0.06-0.25) | 22 (10-38) | 0.59 (0.26-1.05) |
| Tuvalu | 0 (0-0) | 0.33 (0.12-0.65) | 0 (0-0) | 0.78 (0.28-1.55) |
| Uganda | 10 (3-21) | 0.17 (0.05-0.37) | 68 (26-131) | 0.55 (0.2-1.06) |
| Ukraine | 407 (179-698) | 0.56 (0.25-0.96) | 787 (343-1364) | 1.04 (0.45-1.81) |
| United Arab Emirates | 5 (2-12) | 1.59 (0.46-3.38) | 158 (49-329) | 4.77 (1.56-9.79) |
| United Kingdom | 1081 (472-1900) | 1.15 (0.5-2.03) | 2390 (1076-4166) | 1.8 (0.81-3.13) |
| United Republic of Tanzania | 16 (6-31) | 0.16 (0.06-0.32) | 84 (35-158) | 0.38 (0.16-0.71) |
| United States of America | 4325 (1909-7561) | 1.32 (0.58-2.32) | 12186 (5661-20598) | 2.11 (0.98-3.56) |
| United States Virgin Islands | 0 (0-1) | 0.6 (0.26-1.07) | 4 (2-8) | 2.41 (1.09-4.19) |
| Uruguay | 31 (13-57) | 0.79 (0.32-1.45) | 113 (51-197) | 1.99 (0.91-3.48) |
| Uzbekistan | 15 (6-29) | 0.14 (0.05-0.27) | 126 (56-223) | 0.78 (0.35-1.37) |
| Vanuatu | 0 (0-0) | 0.23 (0.08-0.48) | 1 (0-2) | 0.55 (0.2-1.12) |
| Venezuela (Bolivarian Republic of) | 20 (8-35) | 0.22 (0.09-0.4) | 294 (122-576) | 1.04 (0.43-2.03) |
| Viet Nam | 42 (11-93) | 0.11 (0.03-0.25) | 430 (143-855) | 0.53 (0.17-1.06) |
| Yemen | 4 (1-9) | 0.09 (0.02-0.22) | 31 (11-64) | 0.26 (0.09-0.54) |
| Zambia | 6 (2-13) | 0.26 (0.08-0.53) | 32 (13-62) | 0.54 (0.22-1.07) |
| Zimbabwe | 19 (8-35) | 0.55 (0.21-1.04) | 75 (32-138) | 1.21 (0.5-2.27) |

ASMR: age-standardized mortality rate

**Table S2** The DALYs and age-standardized DALY rate of pancreatic cancer attributable to metabolic risk among in countries/territories, 1990 and 2019

| Country/region | DALYs in 1990 | Age-standardized DALY rate in 1990 | DALYs in 2019 | Age-standardized DALY rate in 2019 |
| --- | --- | --- | --- | --- |
| Afghanistan | 334 (105-795) | 4.49 (1.42-10.8) | 1325 (489-2942) | 10.43 (3.88-22.81) |
| Albania | 161 (63-300) | 7.61 (3.07-14.15) | 803 (326-1497) | 18.69 (7.57-34.78) |
| Algeria | 708 (301-1322) | 5.73 (2.36-10.7) | 5967 (2654-10614) | 17.66 (7.83-31.48) |
| American Samoa | 4 (2-7) | 16.87 (7.48-29.84) | 17 (8-29) | 33.67 (15.08-58.02) |
| Andorra | 12 (4-24) | 21.34 (8-41.46) | 42 (18-78) | 30.32 (12.51-55.84) |
| Angola | 145 (37-343) | 3.71 (0.96-8.65) | 976 (368-1957) | 8.5 (3.22-16.94) |
| Antigua and Barbuda | 2 (1-4) | 4.14 (1.64-7.81) | 25 (11-46) | 24.42 (10.33-44.71) |
| Argentina | 6792 (2694-12312) | 20.72 (8.18-37.66) | 20412 (8834-36257) | 38.03 (16.54-67.57) |
| Armenia | 439 (180-788) | 15.6 (6.44-28.05) | 1556 (684-2697) | 36.98 (16.25-64.09) |
| Australia | 3288 (1438-5685) | 16.8 (7.3-29.01) | 10383 (4672-17572) | 25.58 (11.41-43.19) |
| Austria | 2513 (1067-4532) | 21.65 (9.19-39.16) | 5182 (2267-9156) | 30.25 (13.21-52.89) |
| Azerbaijan | 427 (180-771) | 8.23 (3.51-14.54) | 2556 (1111-4457) | 25.87 (11.23-45.28) |
| Bahamas | 10 (4-17) | 6.24 (2.63-10.88) | 79 (34-144) | 19.43 (8.48-35.7) |
| Bahrain | 50 (22-88) | 28.97 (12.9-52.41) | 410 (181-747) | 42.58 (18.5-76.65) |
| Bangladesh | 775 (172-1964) | 1.71 (0.37-4.38) | 5628 (1674-13263) | 4.31 (1.27-10.11) |
| Barbados | 14 (6-24) | 4.97 (2.28-8.64) | 160 (70-281) | 32.32 (14.33-56.63) |
| Belarus | 1526 (659-2672) | 11.59 (5.02-20.33) | 3229 (1375-5736) | 20.61 (8.69-36.9) |
| Belgium | 2935 (1243-5358) | 19.1 (8.08-34.87) | 5762 (2462-10392) | 26.45 (11.38-47.72) |
| Belize | 4 (2-7) | 4.02 (1.71-7.24) | 73 (34-128) | 25.39 (11.81-44.91) |
| Benin | 75 (28-149) | 3.82 (1.41-7.54) | 677 (279-1260) | 13.98 (5.69-26.32) |
| Bermuda | 5 (2-9) | 8.56 (3.95-14.63) | 42 (19-75) | 32.94 (14.57-58.2) |
| Bhutan | 5 (1-13) | 2.07 (0.49-5.28) | 47 (15-103) | 8.29 (2.69-18.63) |
| Bolivia (Plurinational State of) | 190 (80-371) | 5.86 (2.4-11.42) | 1611 (675-3037) | 18.05 (7.48-34.3) |
| Bosnia and Herzegovina | 838 (361-1502) | 20.05 (8.57-36) | 2513 (1096-4705) | 41.33 (17.97-77.64) |
| Botswana | 43 (16-87) | 7.69 (2.73-15.27) | 386 (171-704) | 28.46 (12.73-52) |
| Brazil | 13112 (5526-23555) | 14.64 (6.13-26.37) | 51874 (23447-89038) | 21.72 (9.75-37.27) |
| Brunei Darussalam | 24 (7-47) | 26.25 (8-51.31) | 115 (44-215) | 40.09 (14.59-74.78) |
| Bulgaria | 2831 (1198-4835) | 22.08 (9.34-37.99) | 5732 (2455-10397) | 42.37 (18.15-75.84) |
| Burkina Faso | 110 (33-235) | 2.56 (0.74-5.53) | 769 (313-1504) | 8.61 (3.44-17.09) |
| Burundi | 73 (23-155) | 3.12 (0.94-6.54) | 176 (58-388) | 3.85 (1.25-8.49) |
| Cabo Verde | 5 (2-10) | 2.2 (0.85-4.2) | 141 (61-258) | 34.31 (14.62-63.08) |
| Cambodia | 110 (31-253) | 2.48 (0.69-5.74) | 1082 (383-2162) | 9.21 (3.2-18.24) |
| Cameroon | 368 (153-668) | 8.15 (3.31-15) | 2915 (1169-5503) | 24.54 (9.88-46.34) |
| Canada | 6123 (2651-10674) | 18.87 (8.07-32.92) | 17524 (8140-29676) | 26 (12.12-44.31) |
| Central African Republic | 47 (13-108) | 3.83 (1.07-8.96) | 122 (35-288) | 5.31 (1.54-12.21) |
| Chad | 50 (15-108) | 1.78 (0.52-3.86) | 319 (116-639) | 5.9 (2.12-11.9) |
| Chile | 1859 (814-3228) | 18.57 (8.13-32.21) | 6997 (3197-12091) | 28.9 (13.22-50.03) |
| China | 48675 (14363-101817) | 5.56 (1.64-11.65) | 275175 (101088-535386) | 13.27 (4.87-25.73) |
| Colombia | 2805 (1155-5070) | 16.2 (6.57-29.41) | 8981 (3816-17047) | 17.05 (7.26-32.37) |
| Comoros | 9 (3-19) | 4.06 (1.31-8.59) | 38 (15-73) | 7.7 (3.08-14.74) |
| Congo | 97 (31-206) | 8.73 (2.87-18.41) | 463 (172-935) | 17.05 (6.33-34.37) |
| Cook Islands | 2 (1-3) | 14.12 (5.75-26.28) | 7 (3-12) | 26.28 (11.63-47.19) |
| Costa Rica | 165 (70-295) | 9.58 (3.99-17.22) | 1381 (581-2591) | 26.81 (11.31-50.26) |
| Croatia | 1661 (714-2866) | 25.52 (11.04-44.14) | 2999 (1332-5327) | 35.03 (15.12-62.54) |
| Cuba | 531 (231-956) | 5.17 (2.25-9.31) | 4408 (1963-7809) | 23.49 (10.45-41.76) |
| Cyprus | 102 (38-191) | 12.23 (4.62-22.95) | 514 (205-941) | 26.08 (10.64-47.73) |
| Czechia | 5656 (2469-9842) | 40.96 (17.68-70.84) | 11778 (5297-21070) | 56.78 (25.63-101.89) |
| Côte d'Ivoire | 197 (74-388) | 4.87 (1.79-9.66) | 1501 (608-2868) | 14.26 (5.75-27.29) |
| Democratic People's Republic of Korea | 669 (178-1519) | 3.92 (1.05-8.77) | 2310 (669-4993) | 7.04 (2.05-15.18) |
| Democratic Republic of the Congo | 695 (243-1433) | 4.32 (1.5-8.94) | 2087 (745-4180) | 5.79 (2.03-11.74) |
| Denmark | 1068 (457-1914) | 13.63 (5.83-24.5) | 3012 (1311-5410) | 26.79 (11.53-48.36) |
| Djibouti | 4 (1-9) | 3.09 (0.91-6.71) | 54 (20-112) | 8.8 (3.16-18.58) |
| Dominica | 4 (2-8) | 6.11 (2.69-10.79) | 35 (15-64) | 39.23 (16.88-71.08) |
| Dominican Republic | 97 (38-181) | 2.55 (1-4.8) | 1026 (403-1997) | 10.87 (4.24-21.11) |
| Ecuador | 275 (129-463) | 5.09 (2.36-8.64) | 3240 (1460-5732) | 21.4 (9.64-37.86) |
| Egypt | 1598 (675-2873) | 5.12 (2.17-9.22) | 13196 (5296-23985) | 19.05 (7.84-34.53) |
| El Salvador | 130 (54-234) | 4.42 (1.82-7.96) | 1199 (493-2277) | 20.59 (8.47-39.11) |
| Equatorial Guinea | 7 (2-17) | 3.4 (0.9-8.42) | 105 (39-207) | 21.58 (7.98-42.31) |
| Eritrea | 22 (6-49) | 2.19 (0.62-4.9) | 159 (54-328) | 6.03 (2.08-12.33) |
| Estonia | 443 (200-758) | 21.58 (9.76-36.85) | 829 (381-1432) | 33.18 (14.99-57.53) |
| Eswatini | 40 (17-74) | 13.75 (5.9-25.68) | 188 (77-362) | 32.46 (13.57-62.21) |
| Ethiopia | 318 (71-843) | 1.61 (0.36-4.26) | 1026 (343-2235) | 2.53 (0.84-5.66) |
| Fiji | 40 (17-77) | 11 (4.36-21.28) | 191 (82-356) | 24.87 (10.73-45.96) |
| Finland | 1958 (844-3438) | 27.42 (11.82-48.27) | 4203 (1805-7593) | 35.19 (15.18-63.2) |
| France | 11083 (4458-20107) | 13.86 (5.55-25.28) | 29104 (12642-51444) | 22.79 (9.88-40.7) |
| Gabon | 55 (19-111) | 9.53 (3.3-19.33) | 322 (128-642) | 29.78 (11.84-59.14) |
| Gambia | 8 (3-18) | 2.37 (0.84-5.04) | 84 (34-168) | 8.92 (3.52-17.73) |
| Georgia | 670 (278-1187) | 10.5 (4.39-18.51) | 1426 (596-2586) | 24.92 (10.52-45.11) |
| Germany | 30217 (12951-53428) | 24.04 (10.29-42.52) | 72625 (31579-127700) | 39.84 (17.33-70.47) |
| Ghana | 485 (177-974) | 7.8 (2.78-15.77) | 5294 (2353-9547) | 32.73 (14.39-59.34) |
| Greece | 3265 (1377-5885) | 21.22 (8.95-38.18) | 6609 (2905-11573) | 30.36 (13.01-53.71) |
| Greenland | 13 (6-23) | 36.03 (15.46-61.68) | 46 (20-81) | 62.45 (28.09-111.2) |
| Grenada | 4 (1-7) | 5.13 (1.94-9.82) | 47 (19-84) | 41.3 (17.08-73.58) |
| Guam | 10 (4-18) | 13.09 (5.49-23.84) | 43 (19-76) | 22.44 (9.68-39.67) |
| Guatemala | 125 (43-246) | 3.44 (1.2-6.73) | 1951 (765-3834) | 17.69 (6.85-34.92) |
| Guinea | 60 (23-116) | 1.79 (0.67-3.48) | 248 (102-469) | 4.54 (1.85-8.63) |
| Guinea-Bissau | 17 (5-37) | 4.06 (1.21-9.12) | 79 (29-161) | 11.1 (4.06-22.32) |
| Guyana | 22 (9-41) | 5.83 (2.33-10.94) | 189 (79-355) | 28.77 (11.94-53.76) |
| Haiti | 97 (31-204) | 2.92 (0.91-6.16) | 592 (186-1289) | 8.42 (2.66-18.41) |
| Honduras | 112 (43-216) | 5.54 (2.09-10.63) | 1017 (355-2141) | 16.99 (5.92-35.92) |
| Hungary | 4736 (2106-7982) | 32.25 (14.32-54.58) | 9124 (4158-16144) | 49.31 (22.41-86.59) |
| Iceland | 56 (24-99) | 19.97 (8.79-35.44) | 132 (58-235) | 24.63 (10.97-43.98) |
| India | 11262 (3492-24700) | 2.53 (0.77-5.57) | 101453 (37733-195654) | 8.82 (3.25-16.97) |
| Indonesia | 3010 (931-6252) | 3.01 (0.93-6.28) | 27542 (9673-57776) | 12.37 (4.19-26.42) |
| Iran (Islamic Republic of) | 1349 (564-2611) | 4.99 (2.05-9.75) | 12860 (5735-22171) | 17.61 (7.89-30.5) |
| Iraq | 889 (345-1715) | 11.23 (4.32-21.81) | 6642 (2776-11890) | 27.81 (11.73-49.89) |
| Ireland | 729 (297-1314) | 17.89 (7.23-32.33) | 1993 (883-3559) | 26.71 (11.85-47.93) |
| Israel | 1118 (485-1986) | 22.88 (9.84-40.57) | 3774 (1664-6611) | 33.01 (14.61-57.65) |
| Italy | 20531 (8583-36756) | 23.02 (9.55-41.17) | 41271 (18036-74443) | 30.4 (12.94-54.97) |
| Jamaica | 70 (31-125) | 4.02 (1.79-7.09) | 663 (283-1235) | 22.49 (9.61-41.78) |
| Japan | 26545 (9184-52383) | 15.39 (5.31-30.36) | 56186 (19324-112700) | 16.89 (5.84-33.69) |
| Jordan | 132 (58-233) | 9.71 (4.2-17.28) | 1694 (776-2874) | 25.38 (11.82-43.06) |
| Kazakhstan | 458 (216-771) | 3.61 (1.7-6.1) | 5043 (2319-8606) | 27.79 (12.76-47.57) |
| Kenya | 185 (62-402) | 2.2 (0.72-4.83) | 1848 (792-3436) | 7.91 (3.38-14.86) |
| Kiribati | 3 (1-6) | 8.01 (3.19-15.15) | 10 (4-19) | 13.35 (5.39-25.89) |
| Kuwait | 101 (45-174) | 16.4 (7.31-28.75) | 695 (318-1223) | 27.68 (12.5-48.57) |
| Kyrgyzstan | 256 (113-458) | 8.25 (3.59-14.78) | 687 (302-1218) | 14.27 (6.3-25.48) |
| Lao People's Democratic Republic | 82 (22-190) | 3.88 (1.04-9.12) | 435 (162-836) | 10.03 (3.67-19.42) |
| Latvia | 736 (330-1254) | 20.56 (9.29-35.28) | 1261 (562-2137) | 33.86 (14.94-57.71) |
| Lebanon | 238 (102-426) | 10.17 (4.3-18.43) | 1534 (689-2710) | 29.65 (13.34-52.47) |
| Lesotho | 50 (18-104) | 5.17 (1.88-10.63) | 253 (103-475) | 19.78 (8.09-37.15) |
| Liberia | 64 (26-125) | 5.66 (2.25-11) | 317 (131-607) | 15.58 (6.45-29.9) |
| Libya | 245 (90-490) | 12.98 (4.79-26.01) | 1877 (812-3478) | 36.3 (15.53-67.5) |
| Lithuania | 838 (360-1462) | 18.59 (7.95-32.33) | 1484 (658-2591) | 27.7 (12.12-48.57) |
| Luxembourg | 105 (44-187) | 19.39 (8.09-34.47) | 309 (131-560) | 31.47 (13.18-57.18) |
| Madagascar | 127 (43-256) | 2.46 (0.83-4.97) | 494 (189-1002) | 4.43 (1.68-9) |
| Malawi | 114 (35-239) | 3.02 (0.91-6.27) | 506 (199-963) | 7.08 (2.76-13.64) |
| Malaysia | 396 (147-769) | 4.38 (1.55-8.76) | 3325 (1338-6262) | 12.44 (5.01-23.5) |
| Maldives | 4 (1-9) | 4.66 (1.22-10.45) | 31 (12-59) | 10.91 (4.13-20.84) |
| Mali | 118 (38-240) | 2.86 (0.92-5.85) | 649 (244-1300) | 7.59 (2.79-15.27) |
| Malta | 95 (39-178) | 21.91 (8.97-41.06) | 263 (109-485) | 28.71 (11.94-52.83) |
| Marshall Islands | 2 (1-3) | 9.39 (3.24-19.07) | 8 (3-17) | 22.55 (8.02-44.3) |
| Mauritania | 65 (26-127) | 6.41 (2.56-12.39) | 355 (151-628) | 17.19 (7.27-30.49) |
| Mauritius | 88 (35-162) | 11.68 (4.52-21.69) | 436 (181-834) | 24.28 (10.16-46.2) |
| Mexico | 9846 (4345-17084) | 23.14 (10.27-40.24) | 32910 (14796-58024) | 27.81 (12.48-48.79) |
| Micronesia (Federated States of) | 5 (2-10) | 10.33 (4.23-19.67) | 20 (8-39) | 25.87 (10.63-49.91) |
| Monaco | 20 (9-36) | 29.96 (13.03-53.88) | 50 (22-88) | 55.72 (24.37-98.01) |
| Mongolia | 57 (23-107) | 5.33 (2.2-9.85) | 366 (146-681) | 13.98 (5.8-25.44) |
| Montenegro | 161 (68-284) | 25.31 (10.76-44.65) | 471 (209-832) | 47.07 (21.09-82.82) |
| Morocco | 644 (255-1210) | 4.62 (1.82-8.81) | 5010 (2039-9401) | 15.67 (6.29-29.4) |
| Mozambique | 131 (38-277) | 2.32 (0.64-4.96) | 855 (311-1707) | 7.92 (2.84-16.11) |
| Myanmar | 899 (235-2086) | 3.86 (0.99-8.97) | 4926 (1843-9684) | 10.71 (3.9-21.09) |
| Namibia | 30 (12-58) | 4.17 (1.68-8.05) | 169 (71-308) | 12.23 (5.09-22.42) |
| Nauru | 1 (0-1) | 14.74 (6.07-27.67) | 1 (0-3) | 27.73 (10.18-54.86) |
| Nepal | 127 (31-332) | 1.38 (0.33-3.62) | 1552 (473-3516) | 6.97 (2.06-15.97) |
| Netherlands | 3797 (1584-6895) | 19.15 (7.97-34.89) | 9523 (4099-16752) | 28.33 (12.26-49.72) |
| New Zealand | 585 (247-1037) | 14.95 (6.24-26.5) | 1614 (708-2737) | 21.2 (9.27-36) |
| Nicaragua | 100 (37-197) | 6.73 (2.45-13.48) | 921 (379-1657) | 21.34 (8.78-38.32) |
| Niger | 38 (13-81) | 1.36 (0.46-2.94) | 305 (108-655) | 4 (1.39-8.67) |
| Nigeria | 1330 (462-2780) | 3.12 (1.07-6.5) | 7625 (3205-13959) | 9.14 (3.79-16.78) |
| Niue | 0 (0-1) | 15.48 (6.35-28.71) | 1 (0-1) | 36.26 (15.39-65.31) |
| North Macedonia | 407 (177-727) | 21.04 (9.18-37.59) | 1556 (659-2859) | 46.74 (19.83-85.22) |
| Northern Mariana Islands | 3 (1-5) | 13.81 (6-24.79) | 21 (9-35) | 36.31 (16.45-62.12) |
| Norway | 1320 (535-2427) | 19.79 (8.19-36.15) | 2398 (1022-4338) | 25.45 (10.83-45.83) |
| Oman | 36 (13-76) | 5.39 (1.89-11.66) | 457 (201-790) | 27.1 (12.18-46.39) |
| Pakistan | 1505 (443-3205) | 2.66 (0.78-5.69) | 9996 (3692-19383) | 9.05 (3.31-17.61) |
| Palau | 3 (1-6) | 32.12 (13.42-58.42) | 14 (6-24) | 61.27 (28.02-110.19) |
| Palestine | 87 (32-179) | 9.92 (3.64-20.8) | 713 (309-1275) | 29.9 (12.66-53.92) |
| Panama | 90 (31-175) | 6.11 (2.11-11.91) | 743 (310-1429) | 18.03 (7.52-34.62) |
| Papua New Guinea | 62 (18-142) | 3.18 (0.9-7.4) | 321 (104-721) | 6.34 (2-14.21) |
| Paraguay | 115 (48-209) | 5.19 (2.16-9.51) | 1118 (461-2089) | 20.18 (8.36-37.92) |
| Peru | 482 (195-880) | 4.03 (1.62-7.36) | 5066 (2223-9405) | 15.94 (6.99-29.72) |
| Philippines | 1786 (661-3466) | 6.04 (2.16-11.81) | 8437 (3317-15741) | 10.72 (4.17-20.2) |
| Poland | 13312 (5831-22814) | 30.17 (13.22-51.59) | 28612 (12599-50776) | 41.93 (18.34-74.24) |
| Portugal | 2582 (1021-4707) | 18.31 (7.31-33.4) | 5843 (2444-10613) | 25.71 (10.55-46.44) |
| Puerto Rico | 300 (136-517) | 8.22 (3.73-14.18) | 1980 (882-3681) | 29.26 (12.91-54.37) |
| Qatar | 23 (10-44) | 22.73 (9.31-44.31) | 396 (167-717) | 46.86 (21.16-81.82) |
| Republic of Korea | 4638 (1558-9118) | 15.05 (5.13-29.52) | 17259 (6436-32851) | 19.04 (7.09-36.2) |
| Republic of Moldova | 815 (357-1408) | 17.6 (7.8-30.29) | 1671 (734-2891) | 28.89 (12.59-50.38) |
| Romania | 5011 (2100-8761) | 17.29 (7.23-30.37) | 12237 (5337-21708) | 35.01 (14.99-62.25) |
| Russian Federation | 35055 (15809-60203) | 18.94 (8.53-32.54) | 61275 (27607-104230) | 26.17 (11.68-44.52) |
| Rwanda | 123 (38-265) | 4.2 (1.28-8.89) | 418 (160-793) | 6.95 (2.65-13.36) |
| Saint Kitts and Nevis | 3 (1-5) | 7.04 (3.06-12.39) | 27 (11-48) | 39.27 (16.62-70.7) |
| Saint Lucia | 6 (3-12) | 7.23 (2.9-13.17) | 77 (33-138) | 35.54 (15.33-64.07) |
| Saint Vincent and the Grenadines | 4 (1-7) | 5.4 (2.03-10.01) | 39 (16-70) | 28.38 (11.86-50.75) |
| Samoa | 15 (6-27) | 16.09 (6.64-29.26) | 32 (13-60) | 20.98 (8.63-39.81) |
| San Marino | 7 (3-12) | 20.09 (7.98-36.48) | 21 (8-42) | 34.01 (12.91-69.04) |
| Sao Tome and Principe | 2 (1-3) | 2.8 (1.08-5.29) | 10 (4-20) | 9.53 (3.72-18.9) |
| Saudi Arabia | 357 (137-702) | 6 (2.22-12.1) | 5492 (2500-9438) | 27.08 (12.57-45.61) |
| Senegal | 163 (60-325) | 5.11 (1.88-10.24) | 1058 (430-1954) | 14.42 (5.89-26.62) |
| Serbia | 2635 (1144-4647) | 22.26 (9.75-39.29) | 7218 (3103-13049) | 45.36 (19.61-81.32) |
| Seychelles | 7 (3-14) | 13.24 (5.26-24.9) | 35 (14-64) | 31.16 (12.41-57.44) |
| Sierra Leone | 37 (12-77) | 1.95 (0.62-4.03) | 238 (94-458) | 6.74 (2.63-13.01) |
| Singapore | 294 (98-583) | 13.25 (4.42-26.39) | 1165 (470-2197) | 14.66 (5.9-27.81) |
| Slovakia | 1529 (659-2682) | 25.32 (10.85-44.53) | 3503 (1555-6214) | 37.85 (16.66-67.12) |
| Slovenia | 600 (258-1087) | 24.44 (10.49-44.24) | 1356 (585-2428) | 32.61 (13.95-58.16) |
| Solomon Islands | 7 (2-16) | 4.76 (1.47-10.41) | 45 (16-91) | 12.76 (4.51-25.81) |
| Somalia | 57 (14-147) | 2.26 (0.53-5.92) | 181 (42-483) | 2.71 (0.64-7.23) |
| South Africa | 2935 (1317-5181) | 14.01 (6.25-25.1) | 10901 (5140-18554) | 24.38 (11.65-41.26) |
| South Sudan | 95 (33-195) | 3.97 (1.39-8.22) | 256 (94-526) | 6.61 (2.42-13.54) |
| Spain | 11138 (4815-19965) | 20.26 (8.72-36.06) | 25323 (11402-44615) | 28.39 (12.79-49.86) |
| Sri Lanka | 400 (141-800) | 3.81 (1.34-7.64) | 2961 (1154-6008) | 11.41 (4.42-23.06) |
| Sudan | 274 (92-648) | 2.91 (0.95-6.91) | 2715 (1027-5961) | 14.48 (5.33-31.95) |
| Suriname | 14 (6-25) | 5.33 (2.25-9.83) | 203 (87-370) | 33 (13.98-60.13) |
| Sweden | 3234 (1383-5747) | 22.1 (9.51-39.23) | 5359 (2348-9334) | 26.57 (11.79-46.13) |
| Switzerland | 1502 (634-2715) | 14.68 (6.16-26.57) | 4085 (1760-7357) | 24.27 (10.51-43.4) |
| Syrian Arab Republic | 287 (116-542) | 5.3 (2.11-10.13) | 1919 (783-3653) | 14.98 (6.1-28.46) |
| Tajikistan | 147 (52-313) | 5.33 (1.84-11.46) | 661 (255-1296) | 13.89 (5.15-27.01) |
| Thailand | 1916 (661-3804) | 5.31 (1.78-10.67) | 11263 (4432-22059) | 10.96 (4.28-21.68) |
| Timor-Leste | 5 (1-12) | 1.97 (0.53-4.7) | 53 (16-113) | 6.52 (1.91-13.88) |
| Togo | 38 (14-76) | 3.12 (1.14-6.27) | 376 (154-715) | 10.38 (4.2-20.03) |
| Tokelau | 0 (0-0) | 8.22 (3.26-15.1) | 0 (0-1) | 20.17 (8.22-38.53) |
| Tonga | 6 (2-11) | 10.07 (3.97-19.22) | 16 (7-31) | 20.71 (8.38-38.65) |
| Trinidad and Tobago | 51 (22-90) | 6 (2.55-10.6) | 552 (234-1040) | 29.2 (12.38-55.21) |
| Tunisia | 300 (116-573) | 5.85 (2.28-11.27) | 2158 (909-4108) | 16.85 (7.11-31.91) |
| Turkey | 5759 (2487-10383) | 15.53 (6.72-28.18) | 28824 (12354-51321) | 32.23 (13.97-57.16) |
| Turkmenistan | 64 (28-111) | 3.28 (1.45-5.66) | 556 (244-988) | 13.6 (6.07-23.93) |
| Tuvalu | 1 (0-1) | 7.6 (2.84-14.99) | 2 (1-4) | 17.81 (6.7-35.26) |
| Uganda | 225 (65-477) | 3.53 (1.02-7.52) | 1667 (648-3224) | 11.78 (4.5-22.78) |
| Ukraine | 9996 (4350-17199) | 13.8 (6.02-23.85) | 19422 (8366-34112) | 26.77 (11.36-47.29) |
| United Arab Emirates | 161 (51-349) | 34.47 (10.11-73.74) | 5294 (1629-11164) | 104.37 (34.08-213.35) |
| United Kingdom | 20681 (9091-36422) | 23.18 (10.15-40.87) | 41915 (18677-72452) | 34.47 (15.52-59.84) |
| United Republic of Tanzania | 385 (143-749) | 3.5 (1.32-6.77) | 2050 (850-3844) | 8.24 (3.43-15.4) |
| United States of America | 88614 (39823-153677) | 28.5 (12.84-49.4) | 239345 (113189-400294) | 43.49 (20.62-72.7) |
| United States Virgin Islands | 11 (5-19) | 12.54 (5.5-22.26) | 95 (43-165) | 50.65 (22.91-88.77) |
| Uruguay | 658 (267-1232) | 16.89 (6.82-31.58) | 2123 (965-3692) | 40.97 (18.86-71.18) |
| Uzbekistan | 367 (142-698) | 3.16 (1.23-5.99) | 3377 (1488-5964) | 16.02 (7.07-28.15) |
| Vanuatu | 3 (1-7) | 5.11 (1.83-10.7) | 23 (9-46) | 12.81 (4.75-25.63) |
| Venezuela (Bolivarian Republic of) | 455 (195-806) | 4.72 (1.98-8.41) | 6563 (2708-12770) | 22.22 (9.22-43.2) |
| Viet Nam | 878 (235-1920) | 2.22 (0.59-4.84) | 8883 (2959-17752) | 10.05 (3.33-20.17) |
| Yemen | 97 (28-236) | 1.93 (0.56-4.74) | 765 (291-1539) | 5.68 (2.13-11.65) |
| Zambia | 161 (56-324) | 5.58 (1.9-11.18) | 817 (327-1607) | 11.92 (4.78-23.35) |
| Zimbabwe | 441 (187-801) | 11.02 (4.56-20.26) | 1842 (800-3385) | 26.16 (11.07-48.03) |

DALY: disability-adjusted life-year
